# Effectiveness of interventions for improving educational outcomes for people with disabilities in low‐ and middle‐income countries: A systematic review

**DOI:** 10.1002/cl2.70016

**Published:** 2025-02-06

**Authors:** Xanthe Hunt, Ashrita Saran, Howard White, Hannah Kuper

**Affiliations:** ^1^ Department of Global Health Institute for Life Course Health Research Stellenbosch University Cape Town South Africa; ^2^ Campbell South Asia New Delhi India; ^3^ Global Development Network New Delhi India; ^4^ London School of Hygiene and Tropical Medicine, International Centre for Evidence in Disability London UK

**Keywords:** disability, education, inclusion, LMIC, school

## Abstract

**Background:**

People with disabilities are consistently falling behind in educational outcomes compared to their peers without disabilities, whether measured in terms of school enrolment, school completion, mean years of schooling, or literacy levels. These inequalities in education contribute to people with disabilities being less likely to achieve employment, or earn as much if they are employed, as people without disabilities. Evidence suggests that the gap in educational attainment for people with and without disabilities is greatest in low‐ and middle‐income countries (LMICs). Exclusion of people with disabilities from mainstream education, and low rates of participation in education of any kind, are important issues for global equity. Interventions which might have a positive impact include those that improve educational outcomes for people with disabilities, whether delivered in specialist or inclusive education settings. Such interventions involve a wide range of initiatives, from those focused on the individual level – such as teaching assistance to make mainstream classes more accessible to children with specific learning needs – to those which address policy or advocacy.

**Objectives:**

The objectives of this review were to answer the following research questions: (1) What is the nature of the interventions used to support education for people with disabilities in LMICs? (2) What is the size and quality of the evidence base of the effectiveness of interventions to improve educational outcomes for people with disabilities in LMICs? (3) What works to improve educational outcomes for people with disabilities in LMICs? (4) Which interventions appear to be most effective for different types of disability? (5) What are the barriers and facilitators to the improvement of educational outcomes for people with disabilities? (6) Is there evidence of cumulative effects of interventions?

**Search Methods:**

The search for studies followed two steps. Firstly, we conducted an electronic search of databases and sector‐specific websites. Then, after initial screening, we examined the reference lists of all identified reviews and screened the cited studies for inclusion. We also conducted a forward search and an ancestral search. No restrictions in terms of date or format were placed on the search, but only English‐language publications were eligible for inclusion.

**Selection Criteria:**

In our review, we included studies on the basis that they were able to detect intervention impact. Descriptive studies of various designs and methodologies were not included. We also excluded any study with a sample size of fewer than five participants. We included studies which examined the impact of interventions for people with disabilities living in LMICs. There were no restrictions on comparators/comparison groups in included studies. However, to be eligible for inclusion, a study needed to have both an eligible intervention and an eligible outcome. Any duration of follow‐up was eligible for inclusion.

**Data Collection and Analysis:**

We used EppiReviewer for bibliographic management, screening, coding, and data synthesis. Eligibility was assessed using a predesigned form based on the inclusion criteria developed by the authors. We piloted all coding sheets with at least five studies before use. The form allowed for coding of multiple intervention domains and multiple outcomes domains. The entire screening process was reported using a PRISMA flow chart. We screened all unique references from our search title and abstract, with two independent reviewers determining relevance, and repeated this process for full texts. Data was extracted from studies according to a coding sheet. Coding included: (1) extraction of basic study characteristics, (2) a narrative summary of procedures and findings (including recording of iatrogenic effects), (3) a summary of findings/results table, (4) an assessment of confidence in study findings, and (5) creation of a forest plot of effect sizes. A third data collector, a research associate, checked the results of this process. Confidence in study findings was assessed using a standardised tool. All coding categories were not mutually exclusive and so multiple coding was done where an intervention covered more than one category of intervention.

**Main Results:**

Twenty‐eight studies were included in this review. Most studies (*n* = 25) targeted children with disabilities. Only two studies directly targeted family members, and the remaining three focused on service providers. Individuals with intellectual or learning and developmental impairments were most frequently targeted by interventions (*n* = 17). The category of interventions most represented across studies was ‘Educational attainment support’, for instance, a reading comprehension intervention that combined strategy instruction (graphic organisers, visual displays, mnemonic illustrations, computer exercises, predicting, inference, text structure awareness, main idea identification, summarisation, and questioning) for children with dyslexia. The second most common category of intervention was ‘Accessible learning environments’, for instance, programmes which aimed to improve social skills or to reduce rates of victimisation of children with disabilities in schools. Regarding intervention effects, included studies concerned with ‘Conditions for inclusion of people with disabilities in education’ showed a moderately significant effect, and one study concerned with teacher knowledge showed a significant effect size. Among the 18 studies included in the analysis of intervention effects on ‘Skills for learning’, 12 interventions had a significant effect. When considering the effect of interventions on different outcomes, we see that the effect on literacy, cognitive skills, handwriting, and numeracy are significant. All these effects are large but are based on a low number of studies. The studies concerned with speech and school behaviour show no significant effect of intervention. Across studies, heterogeneity is high, and risk of publication bias varies but was frequently high. All but one study received an overall rating of low confidence in study findings. However, this lack of confidence across studies was largely due to the use of low‐rigour study designs and was not always reflective of multiple points of weakness within a given study.

**Authors' Conclusions:**

Children with disabilities fall behind in educational outcomes as the current school systems are not set up to teach children with different impairment types. There is no one ‘magic bullet’ intervention which can equalise health outcomes for this group. A twin‐track approach is needed, which both addresses the specific needs of children with disabilities but also ensures that they are included in mainstream activities (e.g., through improving the skills of teachers and accessibility of the classroom). However, currently most interventions included in this systematic review targeted individual children with disabilities in an attempt to improve their functioning, skills, and competencies, but did not focus on mainstreaming these children into the school by system‐level or school‐level changes. Consequently, a focus on evaluation of interventions which target not just the individual with a disability but also their broader environment, are needed.

## 1 PLAIN LANGUAGE SUMMARY

### 1.1 Title

A range of individual‐level interventions work to improve educational outcomes among people with disabilities in low‐ and middle‐income countries, but there is little research on systemic or school‐level change.

### 1.2 The review in brief

A range of interventions work to improve educational outcomes among people with disabilities, but these are mostly targeted at people with disabilities. More systemic approaches are needed to improve rates of access to, and likelihood of successful engagement in, education.

### 1.3 What is this review about?

People with disabilities often fall behind in education compared to their peers without disabilities. This is true for enrolment, retention, and completion of education. These inequalities in education contribute to people with disabilities being less likely to gain employment and earn as much as people without disabilities. The gap in educational attainment between people with and without disabilities is largest in LMICs. There is an urgent need to address barriers to the inclusion of people with disabilities, and test approaches to improve their access to and the success in formal and non‐formal educational programmes.

### 1.4 What is the aim of this review?

For this Campbell systematic review, we wanted to analyse and then summarise the findings from research studies that evaluated interventions to improve the educational outcomes of people with disabilities in LMICs.

### 1.5 What are the main findings of this review?

The review shares findings from a range of interventions and outcomes that were identified across 28 studies. Most of the studies included were aimed at children with disabilities, with a few targeting family members or service providers. People with intellectual or learning and developmental impairments were most frequently targeted by interventions. Eight of the included studies were from India, four were from Iran, two were from China, two were from South Africa and two were from Egypt. One study from each of the following countries was also included: Brazil, Jordan, Kenya, Lebanon, Romania, Turkey, Malaysia, Nigeria, Uganda, and Zambia. Few studies reported whether their setting was urban or rural. Most commonly, interventions were delivered in classrooms in mainstream or inclusive settings, followed by specialist school and resource rooms of inclusive schools.

The category of interventions most commonly represented across studies were those aimed at providing support for educational attainment, followed by those focused on improving the accessibility of learning environments. Educational attainment (including skills for formal learning in schools and skills for life) was the most commonly reported outcome. This was followed by more accessible learning environments, such as strengthened learning/social environment(s) and improved social inclusion.

All but one study received an overall rating of low confidence in study findings. However, low ratings were mostly due to the use of low‐rigour study designs and was not always reflective of weakness in the actual study. Generally, methodological details were poorly reported.

### 1.6 What do the findings of this review mean?

Many included interventions were effective at improving children's functioning, skills, and competencies, but did not focus on institutional (i.e., systemic or school‐level) changes. In terms of expanding the research evidence available, strong methodological procedures should be followed and reported on to allow for thorough assessment and comparisons across interventions. Where possible, interventions should be evaluated in terms of concrete outcomes like school completion. Larger sample sizes that include and track outcomes for diverse demographic profiles would help to increase the rigour and reliability of findings, as would the use of standardised measures.

It is well established that a twin‐track approach is needed to improve inclusion and outcomes for people with disabilities, meaning a focus both on targeting their specific needs but also ensuring they are included in mainstream activities. However, this review showed that most included interventions tried to improve children's functioning, skills, and competencies, but did not focus on efforts for mainstreaming through institutional (i.e., systemic or school‐level) changes. There is a need for evaluation of interventions which target not only the individual with a disability, but also ensure inclusion in their broader environment(s). Efforts should also be made to integrate measures of disability within mainstream education impact evaluations and other demographic/household surveys that include education outcomes, and existing non‐targeted government programmes should evaluate whether they are effective in improving educational outcomes for people with disabilities.

### 1.7 How up‐to‐date is this review?

The review authors searched for studies up to March 2022. This Campbell Systematic Review was published in February 6, 2025.

## 2 BACKGROUND

### 2.1 The problem, condition, or issue

The main problem addressed by this review is that people with disabilities are less likely to be enroled in school or to progress as well as their peers without disabilities. These inequalities in education contribute to people with disabilities being less likely to achieve employment (Department of Economic and Social Affairs: Disability, no date), or if they are employed, to earn as much as people without disabilities (Equality and Human Rights Commission, 2017).

Despite the lack of comparable data on education for people with disabilities, recent reports (UNESCO, 2020; World Bank, 2019) showed that people with disabilities were consistently falling behind in educational outcomes compared to their peers without disabilities, whether measured in terms of school enrolment, school completion, mean years of schooling, or literacy levels. For instance, UNESCO's 2020 Global Education Monitoring Report (UNESCO, 2020) noted that children with disabilities make up 15% of the out‐of‐school population, and that individuals with a sensory, physical or intellectual disability are two and a half times more likely than non‐disabled individuals never to have been in school (UNESCO, 2020).

Evidence suggests that the gap between educational attainment for people with and without disabilities is greatest in LMICs. In a 2014 study, children with disabilities were found to be 5 to 10 times more likely to be excluded from school than children without disabilities, and children with learning or communication impairments were consistently among the least likely to attend school, particularly in Africa (Kuper et al., 2014). This finding has been supported by subsequent analyses; in 2018, a study by Mizunoya et al. (2018) showed that the disability gap in school attendance was statistically significant in all 15 LMICs the authors examined. In these settings, living with a disability reduced the probability of being in school by a median 30.9% (Mizunoya et al., 2018).

Importantly, Mizunoya et al. (2018) indicated that neither individual nor socio‐economic and household characteristics explained the scale of the disability gap in education. This suggests that there is something in the environment of education – for instance, in the way schools are structured and functioning, the way learning happens, the way teachers and peers interact with children with disabilities, and other factors not captured by demography – is keeping children with disabilities out of school, and as such, unable to achieve positive educational outcomes. Research from LMICs supports this assertion. For instance, evidence from Uganda suggests that barriers in the built environment at schools hinders inclusion (Wapling, 2016). Large class sizes (Hove, 2014; Wapling, 2016) and poor attitudes to educating children with disabilities by mainstream school educators (De Boer et al., 2011) are also reported to limit educational success among children with disabilities in LMICs.

### 2.2 Education for children with disabilities: Specialist or inclusive?

There is an ongoing and important debate around different approaches to providing education for children with disabilities: ‘mainstreaming’ or inclusive education, versus ‘special needs’ or segregated education. Historically, when people with disabilities were granted access to education, that education mostly happened in so‐called ‘special’ schools (hereafter called specialist schools). These were segregated learning environments where only children with disabilities were admitted, and where they would engage in learning separately from children without disabilities.

In the past two decades, there has been a significant shift in this status quo, with a movement from segregated to inclusive education which, in the school context, refers to the process of bringing children with or without special education needs together in the same premises and under the same conditions (Ghergut, 2012). In other words, learning environments where children with disabilities and children without disabilities are educated together. The right to inclusive education was initially noted in the 1994 Salamanca Statement and Framework for Action (UNESCO, 1994). However, it was the 2006 UN Convention on the Rights of Persons with Disabilities (UNCRPD) (UN General Assembly, 2007) which established inclusive education as a legal right, mandating countries to support its achievement.

Inclusive education requires that learning environments, which previously catered only to relatively homogenous groups of students who learned in similar ways, be adapted and resourced to allow the full participation of all pupils, regardless of ability (Ghergut, 2012; Stubbs, 2008). It implies contexts beyond school, and if followed through to fruition, would see people with disabilities included in learning that begins at birth, is lifelong, and includes learning in the home, the community, and in formal, informal and non‐formal situations (Stubbs, 2008).

Inclusive education, in light of the UNCRPD, is a key tenet of education and/or disability policy in a number of countries (Lindsay, 2007). Yet, the ideal of inclusive education in relation to disability is not without its limitations and complexities. In LMICs and poorly resourced contexts, in particular, the lack of experienced teachers, teaching aides in classrooms, high child‐to‐teacher ratios, and poor financing for inclusion can result in people with disabilities being ‘housed’ in a mainstream school, but not truly experiencing or benefitting from inclusion in any meaningful way (Wapling, 2016). Even in well‐resourced settings, achieving the ideals of inclusive education is a human resource‐intensive undertaking, as teachers must address academic needs based on individual ability.

Further, while ‘special education’ has long been criticised as segregationist and discriminatory (Lipsky & Gartner, 1987), children with certain types of impairments may benefit from separate instruction where the environment and educators cater to their specific learning needs. For instance, there are difficulties with social integration, communication, and friendship for children who are Deaf but being educated in mainstream schools (Wolters et al., 2011). These specific circumstances, as well as the slow pace of transformation toward inclusive education in many countries, means that it is important to consider both inclusive and specialist school settings to fully account for the state of education for people with disabilities.

In the context of a systematic review, inclusive education is a thorny issue. Firstly, definitions of what passes as inclusive education differ widely. So‐called inclusive environments range from on the one hand, settings where specialised services for children with disabilities simply do not exist, so these children are absorbed into mainstream classrooms by default, to on the other hand, well‐resourced, integrated classrooms in which children with and without disabilities participate fully in learning activities and are all provided the supports necessary. Secondly, there are numerous and varied models for implementing inclusive education.

These issues of clarity and definition mean that it can be hard for a systematic review to draw meaningful connections and comparisons between different interventions, even when they are called ‘inclusive education interventions’. For the purposes of this review, we define inclusive education broadly, according to the UNICEF (no date) definition:

Inclusive education means all children in the same classrooms, in the same schools. It means real learning opportunities for groups who have traditionally been excluded.

As such, we considered both specialist and inclusive interventions that aimed to improve educational outcomes for people with disabilities.

### 2.3 The significance of this review

The widespread exclusion of people with disabilities from mainstream education and their low rates of participation in education of any kind are important problems. First, people with disabilities have a fundamental right to education. Both the UNCRPD and the United Nations Convention on Rights of the Child recognise the right of persons with disabilities to education and calls on signatory states to facilitate their full and equal participation in education. This exclusion is also a development issue, as the Sustainable Development Goals call for quality education for all, and include a target related to addressing inequitable access to education for people with disabilities. Additionally, there are multiple benefits to the inclusion of children with disabilities in schooling, both in terms of social participation and for the improvement of future employment prospects. Educational inclusion therefore creates positive outcomes for people with disabilities, both financial and non‐financial. There are also numerous benefits to including people with disabilities in lifelong learning, that is, education beyond the school years, including non‐formal education and life skills education. Opportunities before school, such as early childcare and education, are equally important for all individuals, to support optimal childhood and lifelong development. The review focusses on improving education outcomes for people with disabilities. Early childhood development (ECD) to improve development and prevent disability for children in general was therefore not eligible. ECD targeted or including children with disabilities, where results were reported separately for children with disabilities, would be eligible.

To improve educational outcomes for people with disabilities, barriers to inclusion need to be addressed. These barriers operate to produce decreased rates of school attendance, poorer experiences in school and lower educational outcomes. These barriers operate at the level of the system (e.g., lack of policy), school (e.g., lack of accessible infrastructure or skilled teachers), and the family/child (e.g., poor health), as highlighted in UNESCO's 2020 Global Education Monitoring Report (UNESCO, 2020). The report notes, for instance, that policy and legislative barriers are prevalent, with laws in 25% of countries (but over 40% in Asia and in Latin America and the Caribbean) making provisions for education in separate settings, 10% for integration and only 17% for inclusion of children with disabilities in mainstream schools (UNESCO, 2020).

In response to these circumstances, education is considered a core component in the WHO Community Based Rehabilitation (CBR) programme, a comprehensive and multi‐sectoral strategy aimed at equalising opportunities and including people with disabilities in all aspects of community life (WHO, 2010). CBR promotes the equalisation of opportunities between disabled people and people without disabilities, and strives for the widespread inclusion of people with disabilities in all spheres of life (WHO, 2010). As such, the Guidelines see education interventions as key to their multisectoral approach (WHO, no date). Indeed, education is important for a vast number of social, environmental, economic, and human capital development goals. CBR educational guidance documents note the global need to expand and improve the quality, availability, accessibility, and equitability of education for children with disabilities (WHO, 2010). The CBR programme also has an emphasis on early education, lifelong learning, and non‐formal education for people with disabilities.

Although these international directives place obligations on states to respect, protect, and fulfil the right to education of people with disabilities, evidence on which interventions are actually effective for achieving the outlined goals have not been established. Indeed, past evidence syntheses on the topic of education and disability in LMICs have highlighted that very little literature has compared the educational outcomes of disabled people and their non‐disabled peers (Wapling, 2016). Furthermore, a majority of studies have focused on specialist school populations and did not address questions of attendance or attainment (Maulik & Darmstadt, 2007). Consequently, there is a real need to evaluate interventions in the realm of disability and education to determine ‘what works’ to ensure educational inclusion and produce good educational outcomes for people with disabilities.

### 2.4 A note on defining education

Many LMICs are postcolonial, non‐Western contexts. This raised an important issue for this review to address: ‘What do we mean by education?’ In many LMICs, indigenous knowledge has historically been and is still accorded a lower status than institutional knowledge. For instance, low status may be accorded to the oral transmission of intergenerational knowledge about which land is arable and which not, while high status is accorded to a university degree in agriculture. The systematic review format privileges ‘Western’ positivist thought. While we are willing to include studies which explore indigenous knowledge transfer in the context of disability, we are unlikely to find this information by examining published, written literature. As such, we note that the types of education and educational outcomes privileged in this type of inquiry focus on those delivered through formal institutions of learning (e.g., schools, universities, and vocational training centres), as opposed to other, less quantifiable forms of knowledge transfer. This is a limitation of this review.

### 2.5 The intervention

The interventions we considered in this review were those that sought to improve educational outcomes for people with disabilities, whether delivered in specialist or inclusive education settings. Such interventions involve a wide range of initiatives, from those focused on the individual level, such as teaching assistance to make mainstream classes more accessible to children with specific learning needs, to those which are aimed at improving policies or advocacy strategies.

**Figure 1:**
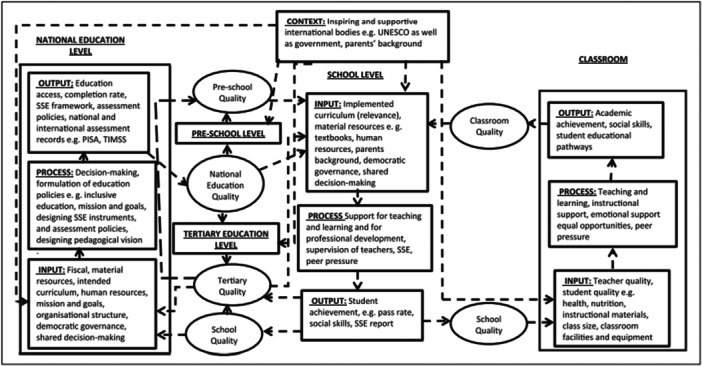
Garira's (2020) unified framework for quality education.

Garira (2020) proposes a unified conceptual framework for quality education in schools. This framework (see Figure 1) highlights the conditions required for quality education at various levels.

Various frameworks have been developed to support the educational inclusion of and outcomes for people with disabilities (Adedeji & Campbell, 2013; Nazar et al., 2018; Vladimirova & Le Blanc, 2016; Walid & Luetz, 2018). It is clear that high‐quality education depends on people with disabilities being included into already high‐quality learning environments (Love & Horn, 2021). As such, improving the quality of global education is a necessary foundation for high‐quality inclusive education. Garira's (2020) framework can thus also assist in conceptualising quality inclusive education.

Taking a systemic approach to quality education, the unified framework is based on an approach where inputs, processes, and outputs can be specified at the national preschool, school, and tertiary levels (Garira, 2020). However, this review focuses on the latter two, and omits ECD interventions.

The interventions of interest in this review are described in Table 1 below. For the purpose of this review, interventions were organised around the education pillar of the CBR matrix, although our taxonomy of interventions was refined based on pilot coding of included papers.

**Table 1:** Types of interventions to improve educational outcomes in people with disabilities.

Intervention domain	Intervention sub‐category	Description	Example
Accessible learning environments	Structural interventions	Interventions that target aspects of the context in which education takes place, such as poverty or poor resourcing of education	Cash transfers to families of children with disabilities
	Learning social environment and social inclusion	Interventions to improve the quality and/or inclusiveness of learning social environments, promote appreciation of diversity, and reduce stigma and discrimination	Teacher trainings on disability awareness and attitudes
	Accessibility of built environment and learning materials (including universal design for learning)	Interventions, including those centred on universal design, to improve physical accessibility of educational spaces	Developing inclusive information technology infrastructure
	Anti‐bullying policies and programmes	Interventions to prevent violence and bullying of students with disabilities, particularly young women and girls	School‐wide anti‐bullying campaigns
	Educational services development	Programmes and policy to provide for the capacity development of teachers (and in certain cases, parents) so that they can educate learners with a wide range of learning needs	Training of teachers in inclusive education practices
	Inclusive education policies	Policies that are developed and implemented in mainstream and special education settings to provide for quality education for people with disabilities	Implementation of inclusive education policy
	Rehabilitation and health services, and assistive technologies	Interventions to make rehabilitation and health services and assistive technologies available to learners with disabilities	Provision of wheelchairs to children with physical disabilities who are of school‐going age
Educational attainment support	Skills for formal education/learning in schools	Interventions to equip people with disabilities with the skills necessary to pursue formal education, including school readiness	Early literacy and numeracy interventions
	Skills for life	Learning‐focused interventions to improve the life skills and living conditions of people with disabilities	Enhancement of attentional capacity or time management
	Education‐related quality of life	Varied programmes that foster improved quality of life for learners with disabilities	Psychosocial support for students with disabilities
Attendance, enrolment, and completion support	Formal enrolment	Interventions to support the enrolment of people with disabilities in formal education (inclusive or specialist)	Community‐based awareness raising of need to enrol children with disabilities in school
	Non‐formal enrolment/participation	Interventions to support the enrolment of people with disabilities in non‐formal education	Community‐based awareness raising about opportunities for education outside of school
	School completion	Interventions to support people with disabilities in completing secondary and higher education, and initiatives to facilitate the acquisition of relevant qualifications by people with disabilities (e.g., high school completion certificates and training certificates)	Tutoring for children with disabilities in final school year
	Transition to higher levels of education	Interventions to support entry into post‐school opportunities on an equal basis with non‐disabled peers	University application support and quotas for disabled students
	Attendance	Programmes to support attendance at school among learners with disabilities	Cash transfers conditional on child's school attendance

### 2.6 How the intervention might work

Interventions which aim to improve educational outcomes for people with disabilities have a variety of foci (Table 1). They include ensuring that:

- Learning environments, including schools, take in all children, including children with disabilities;
- Learning environments, including schools, are inclusive and welcoming and that educators and peers are trained and supported to create an inclusive space for learning by children with disabilities;
- Learning environments, including schools, have adequate infrastructure to be accessible to people with disabilities and provide accessible learning materials;
- Skills for learning are strengthened for people with disabilities;
- People with disabilities are involved in education as role models, educators, policymakers, decision‐makers and contributors;
- The home environments of people with disabilities encourage and support learning;
- Communities are aware that people with disabilities can learn;
- Multisectoral collaboration between the health, education, social and other sectors is established and maintained;
- Rehabilitation and health services, and assistive technologies, are available to learners with disabilities to ensure that they can fully and meaningfully participate in and benefit from, educational opportunities; and
- National policies are comprehensive and facilitate inclusive education.

These different categories of intervention can be conceived of in clusters along a causal chain, that begins with accessible learning environments.


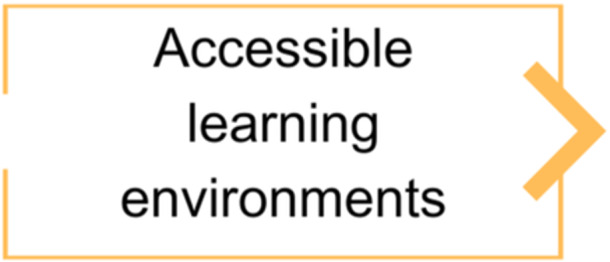


The first set of interventions pertain to (a) addressing the structural forces shaping the context in which education happens, and (b) improving the conditions of a learning environment to better facilitate education for people with disabilities. Structural interventions include those aiming to alleviate poverty, reduce community‐wide stigma against people with disabilities, and/or improve the resources allocated to education at a national or regional level. While many structural interventions do not measure educational outcomes, interventions which did measure outcomes were eligible for inclusion in this review. This is because altering the context in which education happens for people with disabilities in ways that improve educational outcomes is technically an educational intervention. With respect to the immediate conditions in which learning happen, modifications to the school social environment and levels of social inclusion for people with disabilities, accessibility of the built environment and learning materials, educational services development and implementation and resourcing of inclusive education and anti‐bullying policies all contribute to conditions conducive to educational participation by people with disabilities. At this level of intervention, one would also expect to see that rehabilitation services, health services, and assistive technologies are available to learners with disabilities, to ensure that they are able to fully and meaningfully participate in and benefit from educational opportunities.


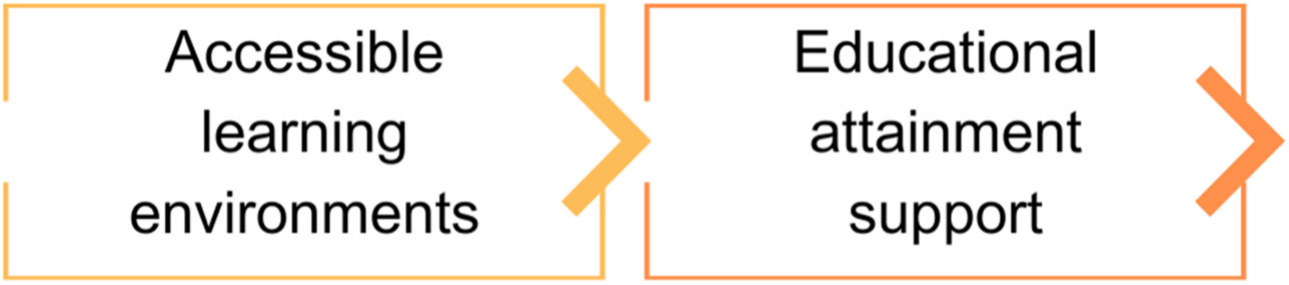


The second cluster of interventions which may improve educational outcomes among people with disabilities are those that aim to equip people with disabilities with the skills necessary to engage in learning. These interventions include a broad range of initiatives in the formal and non‐formal sectors, delivered to individuals of all ages, which aim to equip disabled learners with skills for formal learning (e.g., learning in schools), school readiness, and broader life skills development.


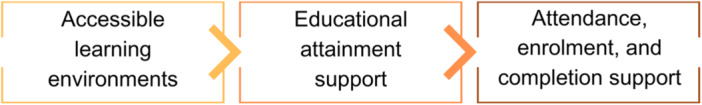


Once learning environments are made accessible, and people with disabilities are equipped with important skills for learning, it is important to deliver interventions aimed at improving attendance in and completion of a variety of kinds of learning. This cluster of interventions includes programming for increased participation by and inclusion of disabled people in formal and non‐formal educational settings. Such interventions seek to improve school completion and attendance among people with disabilities, given that successful educational attainment is predicated on educational participation.

The final cluster of interventions are those which aim to equip people with disabilities with qualifications or improve the throughput rates of people with disabilities at various stages of education, those seeking to improve the education‐related quality of life of disabled students, and interventions aimed at supporting transitions between different levels of education.

At each phase of life, specific programmes for each of these levels of intervention can ensure that people with disabilities are included in mainstream education or have access to specialised educational services when required or desired. These interventions can also help to improve the quality of teaching and the accessibility of learning environments, and to assist disabled people in learning to the best of their ability. Throughout these stages, a supportive legal and policy environment is important, to mandate and monitor inclusive education.

Primary education, which begins at the age of 6 or 7 years and continues into the early teen years, is the pathway to higher levels of education, and socialisation. It is therefore critical for achieving human development goals. Interventions for children with disabilities during this phase can help to create a welcoming, inclusive primary education system where all children are able to fulfil their potential, achieve the best possible educational outcomes, and be well‐positioned to progress to higher forms of education, should they choose. This can be achieved through:

- Programmes to equip families to support their children's involvement in primary education;
- Initiatives aimed at improving the quality of inclusive or specialist primary education;
- Interventions aimed at ensuring that appropriate assistive devices, therapies and other necessary assistance are accessible and available to support education;
- Training and education for teachers so that they feel supported and are confident in their abilities to educate children with disabilities;
- The development of curricula, examination and assessment systems, teaching approaches, and extracurricular activities which are appropriate for children with disabilities;
- The development of local resources for education, including accessible learning materials; and
- Projects which establish and maintain partnerships between relevant stakeholders and involve advocacy at all levels, to ensure that national policies promote primary education for children with disabilities (World Health Organization, 2010).

Secondary and higher education includes both high school and university academic programmes, as well as a variety of technical and vocational educational opportunities. Interventions to support the inclusion of people with disabilities, and their achievement of the best possible educational outcomes, in these levels of education centre on increasing and improving access, participation and achievement for students with disabilities, and ensuring that learning environments are inclusive. Interventions can achieve these aims through:

- Increasing enrolment and retention in and completion of inclusive or specialist secondary and higher education by students with disabilities;
- Helping students with disabilities to access government grants, scholarships and other sources of supportive funding;
- Ensuring that advocacy groups and campaigns for equal access to education exist and are well‐resourced;
- Supporting families and communities to encourage their children with disabilities to pursue secondary and higher education;
- Making sure that secondary and higher education programmes are accessible and inclusive in terms of environment, teaching methods and materials, curricula, extracurricular activities, and assessment and examination systems;
- Promoting learning about diversity and inclusion based on the experiences of (and ideally communicated by) people with disabilities in secondary schools;
- Providing specialist resources and support to enhance inclusion for students with disabilities; and
- Supporting transitions between secondary or higher education programmes into adult life (World Health Organization, 2010).

Finally, other ‘untraditional’ initiatives for learning, such as non‐formal education (sometimes called community education), adult education and lifelong education are also valuable and are not necessarily based in schools or institutions. Such types of education include home‐based learning, government schemes and other local programmes aimed at improving the knowledge and skills of community members. Interventions to improve access to non‐formal education and improve educational outcomes for people with disabilities may focus on:

- Making sure that non‐formal education programmes include people with disabilities and consider their needs during programme planning;
- Actively involving people with disabilities, their family members, disabled people's organisations and parents' associations in decision‐making and implementing non‐formal education programmes; and
- Strengthening social cohesion between students with disabilities and non‐disabled students (World Health Organization, 2010).

Intervention efforts at each of the abovementioned stages of education aim to improve the educational outcomes of people with disabilities through, among other strategies, improving access to education, ensuring that educational opportunities are inclusive, making reasonable accommodations for people with disabilities, and providing specialised supports where necessary. Target outcomes relate to improving environmental conditions, access, attendance, and achievement in education.

### 2.7 Why it is important to do this review

International directives place firm obligations on states to respect, protect, and fulfil the right to education for people with disabilities, as described above. However, evidence on which interventions are effective to achieve the goals they outline have not been established or comprehensively reviewed.

Several relevant Cochrane and Campbell systematic reviews and protocols exist that are relevant to the topic, but none which address the objectives of this review. For instance, in the Cochrane database, one review (Pennington et al., 2018) assessed the effectiveness of parent‐mediated communication interventions for improving the communication skills of preschool children (up to 5 years of age) who have non‐progressive motor disorders. Also from Cochrane, another review (Cogo‐Moreira et al., 2012) considered the evidence on music education as a means for improving reading skills in children and adolescents with dyslexia. A further Cochrane review has been undertaken on task‐oriented interventions for children with developmental co‐ordination disorder (Miyahara et al., 2017). In all cases, however, the scope of these reviews is significantly narrower than our review. In each of these reviews, for example, the focus is on children with particular conditions (i.e., non‐progressive motor disorders, dyslexia and developmental co‐ordination disorder only) and the type of intervention and outcome are limited (parent‐mediated interventions, music interventions and task‐oriented interventions only; communication and reading skills only).

Other rigorous but topic‐specific reviews have been conducted and reported in the peer‐reviewed literature (Buysse & Bailey, 1993; Elbaum et al., 1999; Forlin et al., 2013; Gersten et al., 2001; Hudson et al., 2013; Katz & Mirenda, 2002; Paradise et al., 2009; Pierce et al., 2004; Purdie et al., 2002; Reichrath et al., 2010; Ruijs & Peetsma, 2009; Trout et al., 2003; Wapling, 2016). In one case (Reichrath et al., 2010), interventions in general education for students with disabilities are considered.

However, all of the above reviews are limited in respect of the:

- Geography of research represented, with none being specifically focused on LMICs;
- Type of review (e.g., non‐systematic, narrative, scoping or reviews of reviews) (review type not specified – Forlin et al. (2013); Wapling (2016));
- Impairment type or disabling condition considered [emotional and behavioural disorders only – Trout et al. (2003), Pierce et al. (2004); ADHD only – Purdie et al. (2002); Alzheimer's only Paradise et al. (2009)];
- Eligible outcomes included [reading only – Elbaum et al. (1999), Gersten et al. (2001); development and behaviour only – Buysse and Bailey (1993); academic outcomes only – (Pierce et al., 2004)];
- Other socio‐demographic restrictions, such as location or age of the target population [both children with and without disabilities – Ruijs and Peetsma (2009); Western contexts only Reichrath et al. (2010)];
- Interventions included [inclusive education only – Wapling (2016); Forlin et al. (2013); Katz and Mirenda (2002); Ruijs and Peetsma (2009); teacher‐mediated interventions only – Pierce et al. (2004)]; and/or
- Out of date Buysse and Bailey (1993); Elbaum et al. (1999); Trout et al. (2003).

Finally, White et al. (2018) recently conducted an evidence gap map (EGM) on educational interventions for people with disabilities in LMICs. An EGM can be distinguished from a review in that an EGM is used to identify, map and describe existing evidence of effectiveness, highlight gaps in an evidence base, and sometimes, inform a subsequent systematic review. White et al.'s (2018) EGM discussed impact evaluations and systematic reviews that assessed the effects of interventions for people with disabilities and their families or carers in LMICs and included 46 studies related to education outcomes. Many – but not all – of the same studies were eligible for inclusion in this review, but this review covers an extended time frame compared to the EGM.

## 3 OBJECTIVES

The objectives of this review were to answer the following research questions:

1. What are the characteristics of interventions aimed at supporting education for individuals with disabilities in LMICs?
2. What is the size and quality of the evidence base of the effectiveness of interventions to improve educational outcomes for people with disabilities in LMICs?
3. What works to improve educational outcomes for people with disabilities in LMICs?
4. Which educational interventions appear most effective for different types of disabilities are conducted as standalone interventions?

## 4 METHODS

The protocol for this review was registered by the authors in 2021 (Hunt et al., 2021).

### 4.1 Criteria for considering studies for this review

#### 4.1.1 Types of studies

In our review, we included studies on the basis that they were able to detect intervention impact. This included studies which:

(a) included random allocation of participants;
(b) used a quasi‐random method of participant allocation;
(c) allocated participants according to matched pre‐test and/or relevant demographic characteristics (using observables or propensity scores) and/or a cut‐off on an ordinal or continuous variable (such as in regression discontinuity study designs);
(d) used statistical methods to control for differences between participant groups which existed at baseline (for instance, where multiple regression analysis or instrumental variables regression is used), rather than participants being randomly assigned;
(e) used an interrupted time‐series design, with attempts to detect whether the intervention had an effect which was significantly greater than any underlying trend which would have occurred without intervention over time, using observations at multiple time points before and after the intervention;
(f) used historical controls, with participants who were receiving an intervention being compared to a similar group from the past who had not received the same intervention; and
(g) used a single‐group before‐and‐after design, with observations being made on a group of individuals before and after an intervention, but with no control group.

Descriptive studies of various designs and methodologies (such as qualitative interview studies, single time‐point cross‐sectional surveys, etc.) were not included. We also excluded any study with a sample size of fewer than five participants.

#### 4.1.2 Types of participants

We included studies which examined the impact of interventions for people with disabilities living in LMICs. Population subgroups of interest included: women with disabilities, children with disabilities (particularly vulnerable children with disabilities), people with different impairments, people with disabilities living in conflict and post‐conflict settings, migrants with disabilities, refugees and internally displaced people with disabilities, and ethnic minorities with disabilities. All impairment types were eligible, including physical, mental, intellectual, and sensory impairments.

#### 4.1.3 Types of interventions

There were no restrictions on comparators or comparison groups in the studies that were included. However, to be eligible for inclusion, a study had to have both an eligible intervention *and* an eligible outcome. Eligible interventions were detailed in Table 1.

#### 4.1.4 Types of outcomes and outcome measures

Eligible outcomes, as for interventions, were largely based on the education pillar of the CBR matrix, as shown in Table 2 below, which details the outcomes of interest. All outcomes were considered eligible, regardless of whether they were primary or secondary outcomes of the impact evaluation. The authors of this review have recently undertaken a systematic review of interventions to improve livelihoods among people with disabilities in LMICs. Vocational training programmes were included in that review, where employment/engagement in the labour market was the only outcome, rather than in this review. For a lifelong learning intervention to be eligible for inclusion in this review, it had to have education outcomes other than employment or participation in the labour market.

**Table 2:** Outcome categories and sub‐categories.

Outcome domain	Outcome sub‐category	Description	Example outcome
Accessible learning environments	Strengthened learning environment and improved social inclusion	Learning social environments are inclusive, stigma and discrimination decrease, and people with disabilities are included socially	Improved school climate
	Improved accessibility of built environment and learning materials	Classrooms and educational establishments are physically accessible to learners with disabilities, and learning materials are accessible	Improved accessibility audit scores
	Reduced rates of bullying and victimisation in education setting	Anti‐bullying and anti‐violence interventions are adequately resourced and implemented, and result in reductions in rates of bullying and violence	Reduced rates of violence
	Educational services developed	Teachers, and in some cases parents, acquire appropriate skills to educate learners who have a wide range of learning needs	Improved teacher knowledge, attitudes, and practices regarding disability
	Provision and utilisation of rehabilitation and health services, and assistive technologies	People with disabilities have access to the necessary rehabilitation and health services and assistive technologies necessary to enable their full participation in education	Increased access to assistive devices
Educational attainment	Skills for formal education/learning in schools	People with disabilities acquire skills which are necessary precursors to success in formal education, including improved school readiness	Higher scores on standardised scholastic tests
	Skills for life	People with disabilities make use of youth or adult centred learning opportunities to improve their life skills and living conditions, including through the acquisition of skills for self‐care, self‐management, and integration	Improved capacity for attention
	Education‐related quality of life	Learners with disabilities experience educational opportunities as positive, and as contributing to a good quality of life	Improved education‐related quality of life
Attendance, enrolment, and completion	Formal non‐formal	People with disabilities have resources and support to enrol in quality secondary and higher education in an enabling and supportive environment, and people with disabilities experience equal opportunities to participate in learning opportunities that meet their needs and respect their rights	School enrolment rate increases among disabled children
	Non‐formal enrolment/participation	People with disabilities participate in a variety of non‐formal learning opportunities based on their needs and desires	Improved vocational training enrolment rates for disabled youth
	School completion	People with disabilities have the resources and support to complete quality secondary and higher education in an enabling environment	Qualifications gained
	Attendance	People with disabilities attend secondary and higher education	Improved attendance rates
	Transition to higher levels of education	People with disabilities have access to post‐school options on an equal basis with their peers	Increased university enrolment rate

#### 4.1.5 Duration of follow‐up

Any duration of follow‐up was eligible for inclusion, and studies were coded to analyse the ‘impact trajectory’, that is, how effects varied over time.

#### 4.1.6 Types of settings

All studies needed to originate from an LMIC, as defined by the World Bank. Within these regions, any intervention setting was eligible (e.g., school, home, community).

### 4.2 Search methods for identification of studies

This systematic review was based on an update of searches conducted for an EGM that presented findings on the effectiveness of interventions for people with disabilities in LMICs (Saran et al., 2020). The EGM was commissioned by the United Kingdom's Foreign, Commonwealth & Development Office (FCDO), under its support for the Centre for Excellence for Development Impact and Learning (CEDIL) and the PENDA grant from DFID. For this review, we updated the database search and screened references to identify additional studies. This review was based on the updated searches performed for the EGM in February 2020. The EGM found that with regard to education, few studies reported on the participation of children with disabilities in formal education. The most commonly reported education outcome in their EGM was ‘social and life skills development’ with effects reported from health interventions (rehabilitation and promotion), as well as early child development, and non‐formal education. Our findings differed in this respect, but this difference is largely due to the inclusion and exclusion criteria employed in our systematic review as compared to the EGM. We excluded certain studies where the intervention and outcomes lacked a clear education focus, and so the rehabilitation interventions and social skills development outcomes included under education in Saran et al.'s EGM were excluded here and are instead dealt with in two other systematic reviews on health outcomes and social inclusion outcomes respectively, also by our team. The EGM also did not find many studies conducted with primary and secondary school‐aged participants, which we have included in this review. Again, this is due to different inclusion criteria, partly because the EGM included vocational training with livelihoods outcomes while this review did not.

The search for studies followed three steps.

o First, an electronic search of databases and sector‐specific websites was conducted which was done for the EGM describes above. The list of databases and search term used for the search to update the EGM is described below.
o Open Alex Search: We then utilised eligible studies from the updated EGM that were part of the education review. This led us to conduct an Open Alex search within the EPPI (Evidence for Policy and Practice Information and Coordinating Centre) database, specifically targeting studies published from 2020 to 2022.
o Grey Literature Search: To ensure a thorough review, we also performed searches for grey literature to complement the findings from the Open Alex search for the period between 2020 and 2022.

#### 4.2.1 Electronic searches

We searched the following electronic databases:

- CINAHL.
- ERIC.
- Scopus.
- Web of Science (Social Sciences Citation Index).
- WHO Global Health Index.
- MEDLINE(R).
- Embase Classic + Embase.
- PsycINFO.
- CAB Global Health.

MEDLINE, Embase, PsychINFO, and CAB Global Health were searched through OVID, and ERIC and CINAHL through Ebsco. PubMED was searched through NCBI. We tailored the search strategy for each of the databases (see Annex S1A), but the main search strategy included the following populations, study designs and location terms:

**Population.** (disable* or disabilit* or handicapped) **OR** (physical* or intellectual* or learning or psychiatric* or sensory or motor or neuromotor or cognitive or mental* or developmental or communication or learning) **OR** (cognitive* or learning or mobility or sensory or visual* or vision or sight or hearing or physical* or mental* or intellectual*) adj2 (impair* or disabilit* or disabl* or handicap*) **OR** (communication or language or speech or learning) adj5 (disorder*) **OR** (depression or depressive or anxiety or psychiat* or well‐being or quality of life or self‐esteem or self‐perception) adj2 (impair* or disabilit* or disabl* or handicap*) **OR** mental health **OR** (schizophreni* or psychos* or psychotic or schizoaffective or schizophreniform or dementia* or alzheimer*) adj2 (impair* or disabilit* or disabl* or handicap*) **OR** (mental* or emotional* or psychiatric or neurologic*) adj2 (disorder* or ill or illness*) **OR** (autis* or dyslexi* or Down* syndrome or mongolism or trisomy 21) **OR** (intellectual* or educational* or mental* or psychological* or developmental) adj5 (impair* or retard* or deficien* or disable* or disabili* or handicap* or ill*) **OR** (hearing or acoustic or ear*) adj5 (loss* or impair* or deficien* or disable* or disabili* or handicap* or deaf*) **OR** (visual* or vision or eye* or ocular) adj5 (loss* or impair* or deficien* or disable* or disabili* or handicap* or blind*) **OR** (cerebral pals* or spina bifida or muscular dystroph* or arthriti* or osteogenesis imperfecta or musculoskeletal abnormalit* or musculo‐skeletal abnormalit* or muscular abnormalit* or skeletal abnormalit* or limb abnormalit* or brain injur* or amput* or clubfoot or polio* or paraplegi* or paralys* or paralyz* or hemiplegi* or stroke* or cerebrovascular accident*) adj2 (impair* or disabilit* or disabl* or handicap*) **OR** (physical* adj5 (impair* or deficien* or disable* or disabili* or handicap*) **OR** people with disabilities/or children with disabilities/or people with mental disabilities/or people with physical disabilities/**OR** abnormalities/or exp congenital abnormalities/or exp deformities/or exp disabilities/or exp malformations/**OR** exp mental disorders/or exp mental health/or learning disabilities/or paralysis/or paraparesis/or paraplegia/or poliomyelitis/or hearing impairment/or deafness/or people with hearing impairment/or vision disorders/or blindness/or people with visual impairment/.

**Study design.** (systematic* or synthes*) adj3 (research or evaluation* or finding* or thematic* or report or descriptive or explanatory or narrative or meta* or review* or data or literature or studies or evidence or map or quantitative or study or studies or paper or impact or impacts or effect* or compar*) **OR** (meta regression or meta synth* or meta‐synth* or meta analy* or metaanaly* or meta‐analy* or metanaly* or metaregression or metaregression or methodologic* overview or pool* analys* or pool* data or quantitative* overview or research integration) **OR** (review adj3 (effectiveness or effects or systemat* or synth* or integrat* or map* or methodologic* or quantitative or evidence or literature)) **OR** (meta ethnograph* or meta synthesis or (synthesis and (qualitative literature or qualitative research)) or critical interpretive synthesis or (systematic review and (qualitative research or qualitative literature or qualitative stud*)) or thematic synthesis or framework synthesis or realist review or realist synthesis or qualitative systematic review* or qualitative evidence synthes* or ((quality assessment or critical appraisal or literature search*) and (qualitative research or qualitative literature or qualitative stud*)) or (Noblit and Hare) or meta narrative* or narrative synthesis) **OR** meta‐analysis/or evaluation studies/or qualitative research/or systematic review/**OR** controlled clinical trial/or randomized controlled trial/or equivalence trial/or pragmatic clinical trial/or case‐control studies/or retrospective studies/or cohort studies/or follow‐up studies/or longitudinal studies/or prospective studies/or epidemiologic methods/or epidemiologic studies/or controlled before‐after studies/or cross‐sectional studies/or interrupted time series analysis/or control groups/or cross‐over studies/or double‐blind method/or matched‐pair analysis/or meta‐analysis as topic/or random allocation/or single‐blind method/or retraction of publication/or case reports/**OR** (random or placebo or single blind or double blind or triple blind or cohort or ((case or cohort or follow up or follow‐up) adj2 (control or series or report or study or studies)) or retrospective or (observ adj3 (study or studies))).

**Location.** Developing Countries **OR** Africa/or Asia/or Caribbean/or West Indies/or Middle East/or South America/or Latin America/or Central America/**OR** (Africa or Asia or Caribbean or West Indies or Middle East or South America or Latin America or Central America) **OR** ((developing or less* developed or under developed or underdeveloped or middle income or low* income or underserved or under served or deprived or poor*) adj (countr* or nation? or population? or world or state*)) **OR** ((developing or less* developed or under developed or underdeveloped or middle income or low* income) adj (economy or economies)) **OR** (low* adj (gdp or gnp or gross domestic or gross national)) **OR** (low adj3 middle adj3 countr*) **OR** (lmic or lmics or third world or lami countr*) **OR** transitional countr*.

#### 4.2.2 Searching other resources

As noted, we searched the reference lists of identified recent papers and reviews. We also ensured that we covered the unpublished literature, so as to minimise the risk of publication bias in our review. To this end, we searched the following websites and databases using a tailored keyword search for grey literature (for full list contact authors):

- International Labour Organisation.
- Department for International Development (DfID), including Research for Development (R4D).
- United Nations Educational, Scientific and Cultural Organisation.
- World Health Organization.
- Disability Programme of the United Nations Economic and Social Commission for Asia and the Pacific.
- United States Agency for International Development.
- Dissertation Abstracts, Conference Proceedings and Open Grey.
- Humanity and Inclusion.
- CBM.
- Sightsavers.
- Plan International.

### 4.3 Data collection and analysis

#### 4.3.1 Description of methods used in primary research

**Figure 2:**
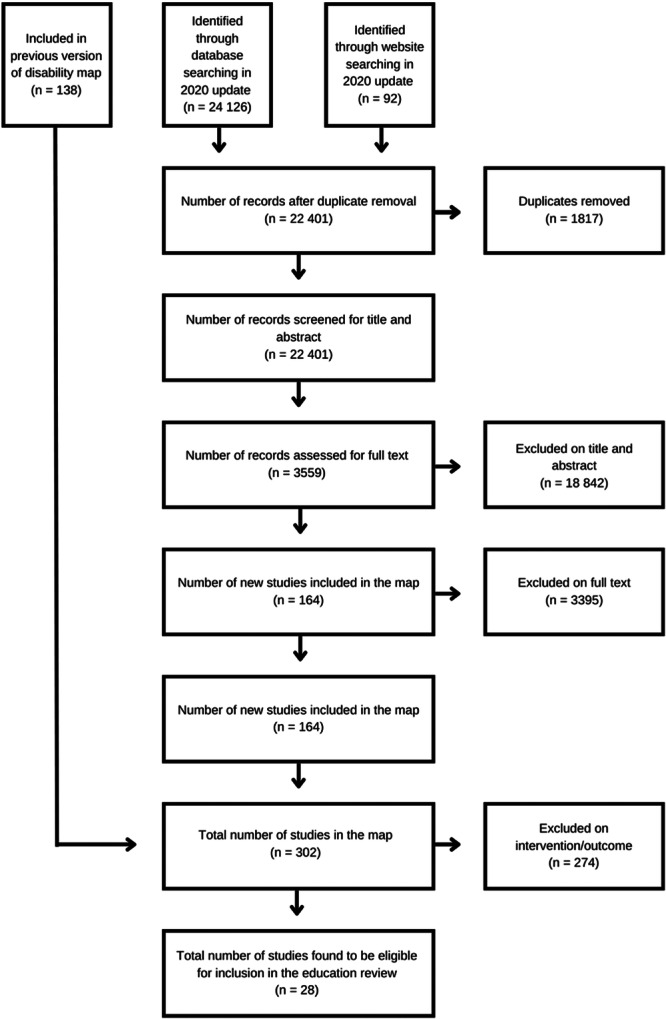
PRISMA flow chart of study screening and selection process.

We used EppiReviewer for bibliographic management, screening, coding, and data synthesis. Eligibility was assessed the inclusion criteria based on PICOS framework as described in detail in the previous section. This form was developed by XH and AS and was reviewed by HK and HW. We piloted all coding sheets with at least five studies before use. The form allowed for coding of multiple intervention domains and multiple outcome domains. Articles excluded at this stage are summarised in the subsection ‘Excluded Studies’ below. The entire screening process was reported using a PRISMA flow chart (see Figure 2 below).

#### 4.3.2 Criteria for determination of independent findings

We did not find multiple publications reporting on the same study.

#### 4.3.3 Selection of studies

We screened all unique references from our search of titles and abstracts. Two independent reviewers determined relevance. If any disagreement arose, it was resolved by HW and HK. A similar process was followed for full texts: the full text of articles which appeared relevant based on title and abstract were screened independently by two independent reviewers, with disagreements resolved by HW and HK. Reviewers demonstrated an 86% rate of agreement.

#### 4.3.4 Data extraction and management

XH and AS worked independently to code the included studies. They extracted data from the studies according to a coding sheet (Annex S1B). A third data collector, a research associate, checked the results of this process. Studies were coded by intervention, outcome(s) and a range of filters (such as age of target population), as well as types of impairments covered. Where appropriate and possible, we extracted the following methodological and quantitative data:

- Study design.
- Analysis method.
- Type of comparison (if relevant).
- External validity.
- Outcome descriptive information.
- Sample size in each intervention group.
- Outcomes means and standard deviations.
- Test statistics (e.g., *t*‐test, *F*‐test, *p*‐values, 95% confidence intervals [CIs]).
- Information on intervention design.
- Confidence in study findings.

As noted, where systematic reviews were discovered by our searches, their reference lists of primary studies were also assessed for eligibility – we have not included summarised findings of the systematic reviews in this review.

#### 4.3.5 Confidence in study findings in included studies

Table 3 presents the tool^1^ we used to assess confidence in study findings. This tool, which the authors are using across a range of disability intervention systematic reviews, contains six criteria:

1. *Study design*: Potential confounders must be considered in assessing the impact of an intervention. Reliable impact evaluations should have either a well‐designed control group, preferably based on random assignment, or an estimation technique which controlled for confounding and the associated possibility of selection bias.
2. *Masking*: Masking, or blinding, is only relevant in randomised controlled trials (RCTs). This procedure helps to limit the biases which can occur if study participants, data collectors or data analysts are aware of the assignment condition of individual participants.
3. *Attrition*: This can be a major source of bias in studies, especially if there is differential attrition between the treatment and comparison group so that the two may no longer be balanced in pre‐intervention characteristics. We applied the standards for acceptable levels of aggregate and differential levels of attrition, according to a set of standards developed by the United States *Institute of Education Sciences What Works Clearing House*.^2^
4. *Clear definition of disability*: For a study to be useful, the study population must be clear, which means that the type and degree of disability should be clearly defined, preferably with reference to a widely used international standard.
5. *Clear definition of outcome measures*: To aid interpretation and reliability of findings and comparability with other studies, outcome measures must be clearly defined. Studies should state the outcomes being used with a definition and the basis on which they are measured, preferably with reference to a widely used international standard.
6. **Baseline balance**: This shows that the treatment and comparison groups are the same at baseline. Lack of balance between groups at baseline can bias the results.

**Table 3:** Study quality assessment criteria.

	Criterion	Low	Medium	High
1	Study design (potential confounders considered)	Before versus after; naïve matching	Instrumental variables (IV) estimation, regression discontinuity design (RDD), propensity score matching (PSM), double difference	RCT, natural experiment
2	Masking (RCTs only)	No mention of masking	Masking for analysis	Masking of data collection (where feasible); masking for analysis
3	Losses to follow up are presented and acceptable	Attrition not reported OR falls well outside *What Works Clearing House* (WWC) acceptable combined levels OR overall attrition >50%	Overall and differential attrition close to WWC combined levels	Overall and differential attrition within WWC combined levels
4	Disability/impairment measure is clearly defined and reliable	No definition	Unclear definition OR Single question item only	Clear definition, for example, Washington Group questions, detailed measure of impairment
5	Outcome measures are clearly defined and reliable	No definition	Unclear definition OR Single question item only	Clear definition using existing measure where possible
6	Baseline balance (N/A for uncontrolled before vs. after)	No baseline balance test (except RCTs) OR reported and significant differences on more than five measures; PSM without establishing common support	Baseline balance test, imbalance on 5 or fewer measures	RCT, RDD
7	Overall confidence in study findings	Low on any item	Medium or high confidence on all items	RCT with high confidence on all items

Confidence in study findings was rated as high, medium, or low, for each of the criteria, based on application of these standards. Overall confidence in study findings was the lowest rating a study achieved across the criteria (e.g., a study receiving low for any criteria would receive an overall rating of low).

#### 4.3.6 Measures of treatment effect

Effect size estimates with 95% CIs) were extracted from included studies. Effect sizes were measured as SMDs with their 95% CIs. In all studies, treatment effects were reported as continuous outcomes. Treatment effects were estimated using SMDs for RCTs and quasi‐experiments with two independent groups by entering the required data into metafor package in R (*M*, SD, *n*). SMDs were calculated using baseline‐adjusted mean differences (i.e., mean change scores) in studies reporting baseline and post‐intervention outcome data. The formulae for these effect sizes are presented in other Campbell review protocols (Sharma Waddington & Cairncross, 2021).

#### 4.3.7 Unit of analysis issues

The unit of analysis of interest to the present review was individual people with disabilities, their caregivers, carers, or those working with them. If a study had more than two intervention arms, we included only intervention and control groups that met the eligibility criteria. Where multi‐arm studies were included, we ensured not to double‐count participants and separately reported eligible interventions and their respective outcomes. No subgroup analyses were conducted.

#### 4.3.8 Dealing with missing data

No included study was eliminated from the analysis due to missing data.

#### 4.3.9 Assessment of reporting biases

Publication bias was assessed visually with funnel plots produced using the *metafor* package in R and tested more formally with Egger's meta‐regression test (Egger et al., 1997). A funnel plot involves plotting the effect size (horizontal axis) against the study's precision (vertical axis). There should be a symmetric distribution of effect sizes between the different studies without publication bias (the vertical line in the centre). In theory, studies with a low degree of precision (at the bottom of the graph) will deviate more from the pooled effect size than studies with a high degree of precision (at the top of the graph), creating a funnel distribution. An asymmetric funnel plot suggests publication bias (Deeks et al., 2005). Egger's test involves a linear regression between the intervention effect estimates and their standard errors weighted by the inverse variance (Egger et al., 1997).

#### 4.3.10 Data synthesis

Data synthesis included: (1) extraction of basic study characteristics; (2) a narrative summary of procedures and findings, including recording of iatrogenic effects; (3) a summary of findings/results table; (4) an assessment of confidence in study findings; and (5) a forest plot of effect sizes. As noted under ‘Assessment of heterogeneity’ above, we also coded effect sizes.

#### 4.3.11 Assessment of heterogeneity

Heterogeneity analysis was conducted for participant, intervention, and outcome characteristics. Because multiple effect sizes may be attributable to sampling error, a random effects model and the associated inverse variance weight at the 95% confidence level was used for all analysis. The random effects model provides for an assumption of population variation from which the sample is drawn and calculates the effect size's impact by estimating that population's parameters. An *I* ^2^ of 0%–40% was interpreted to be low heterogeneity, 41%–80% moderate heterogeneity and 81% and above to mean high heterogeneity (Higgins et al., 2009).

#### 4.3.12 Treatment of qualitative research

We did not include qualitative research in this systematic review.

## 5 RESULTS

### 5.1 Description of studies

#### 5.1.1 Results of the search

The Preferred Reporting Items for Systematic Reviews and Meta‐Analyses (PRISMA) flowchart (Figure 2) outlines the steps we took during the review process. Electronic databases searches yielded 24,126 additional potentially relevant documents for review, while an additional 92 studies were identified from grey literature search, reference and citation searching. The results from all three searches were combined, exported, and deduplicated using the reference management software Eppi reviewer 4 and we identified 1817 duplicates. We reviewed the titles and abstracts of the remaining 22,401 documents to determine potential relevance. We excluded 18,842 due to irrelevance to the review, leaving 3559 articles for full paper review and to determine inclusion in the review. Of these 3559, a further 3395 were excluded, and 164 new studies were deemed relevant for the updated review. These 164 studies were pooled with the 138 studies which had been identified from the previous EGM search, bringing the total count of included studies for the effectiveness map to 302. Of these, 274 were excluded on the basis of ineligible intervention or outcome type, while 28 studies across 15 countries were found to be eligible for inclusion in this education review.

#### 5.1.2 Included studies

Table 4 presents a brief overview of the 28 included studies.

**Table 4:** Intervention and outcome details by study.

Study	Country	Design	Sample size	Intervention	Education outcome domain(s)^a^
Adnams et al. (2007)	South Africa	RCT	65	A language and literacy intervention focused on training in phonological awareness and acquisition of other pre‐ and early literacy skills	Educational attainment
Akbari et al. (2019)	Iran	Controlled before versus after	20	A computer assisted programme for working memory which aimed to improve executive functions and reading performance of students with reading disorder	Educational attainment
Altakhyneh (2019)	Jordan	Controlled before versus after	60	Bruner's approach, developed and supported with the total communication method	Educational attainment
Awada and Gutiérrez‐Colón (2017)	Lebanon	Controlled before versus after	298	A programme delivering combined strategy instruction with the aim of improving the reading comprehension of students with dyslexia	Educational attainment
Carew et al. (2019)	Kenya	Uncontrolled before versus after	130	The Leonard Cheshire Disability inclusive education training programme, designed to increase teaching self‐efficacy, improve inclusive beliefs, attitudes and practices, and reduce concerns around the inclusion of children with disabilities among teachers	Accessible learning environments; attendance and completion
Costescu et al. (2015)	Romania	Controlled before versus after	81	A robot‐assisted reversal learning task to improve cognitive flexibility	Educational attainment
Devries et al. (2018)	Uganda	RCT	3820	The Good School Toolkit, a multi‐component school‐wide intervention which aims to reduce physical violence from peers and school staff toward students with and without disabilities in primary schools	Accessible learning environments
Eissa (2009)	Egypt	Controlled before versus after	67	A programme for school children based on self‐regulated strategy development with a view to improving writing skills	Educational attainment
Hatamizadeh et al. (2020)	Iran	RCT	122	A resilience‐focused intervention focused on building behavioural strengths and addressing difficulties of mainstreamed adolescent students with hearing loss	Educational attainment
Johnson (2018)	India	Controlled before versus after	34	The Cognitive Orientation to Occupational Performance (CO‐OP) programme, which aims to improve handwriting performance in children with Developmental Coordination Disorder (DCD)	Educational attainment
Karahmadi et al. (2014)	Iran	RCT	52	A parent education intervention for children with reading and writing disabilities	Educational attainment
Karande et al. (2007)	India	Uncontrolled before versus after	50	A parent education programme which aimed to educate parents of children with language disorders about the condition and the role of remedial education	Educational attainment
Katongo and Ndhlovu (2015)	Zambia	Controlled before versus after	60	A music intervention which involved ‘drilling songs’ [sic] toward improved speech intelligibility of learners with postlingual hearing impairments	Educational attainment
Kaur et al. (2008)	India	Controlled before versus after	40	Comparative efficacy of multimedia, cognitive, and eclectic strategies on mathematical ability of children with learning disabilities	Educational attainment
Kumar and Chaturvedi (2014)	India	Controlled before versus after	64	A computer‐assisted instruction package (comprising games and simulations) for remedial teaching for children with learning disabilities	Educational attainment
Lal and Bali (2007)	India	Controlled before versus after	30	Visual strategy training using objects, pictures, symbols, and manual signs for the development of communication skills	Educational attainment
Lal (2010)	India	Uncontrolled before versus after	8	The Makaton Vocabulary Language Programme, a system of alternative and augmentative communication (AAC) for children with autism	Educational attainment
Lal and Ganesan (2011)	India	Controlled before versus after	20	A social story intervention to improve self‐management skills in children with autism	Educational attainment
Lee et al. (2019)	China	Uncontrolled before versus after	8	An emotional skills intervention which aimed to improve behavioural and emotional competence and communication for children with autism	Educational attainment
Martin et al. (2001)	China	Uncontrolled before versus after	47	Teacher training techniques for teaching higher‐level critical and creative cognitive strategies to Deaf learners	Educational attainment
Mohammed and Kanpolat (2010)	Egypt	Controlled before versus after	68	Computer‐assisted instruction which aimed to improve child learning outcomes	Educational attainment
Pawar and Mohite (2014)	India	Uncontrolled before versus after	120	A self‐instructional module to improve primary school teachers' knowledge of learning disorders	Accessible learning environments
Rezaiyan et al. (2007)	Iran	Controlled before versus after	60	A computer programme that used a path‐finding game to improve attentional capacity among people with intellectual disabilities	Educational attainment
Thai and Mohd Yasin (2016)	Malaysia	Controlled before versus after	70	The Magic Finger Teaching Method, which entails manipulative techniques using the hands as well as the active involvement of the students in performing arithmetic	Educational attainment
Twilhaar (2012)	South Africa	Uncontrolled before versus after	36	A Conductive Education (CE) intervention utilising a holistic educational system to teach and motivate children with cerebral palsy to participate more in various domains	Educational attainment
Ugwuanyi and Adaka (2015)	Nigeria	Uncontrolled before versus after	33	Auditory training to improve reading comprehension among children with hearing impairments	Educational attainment
Valentini and Rudisill (2004)	Brazil	Controlled before versus after	104	An inclusive mastery climate intervention including activities organised around the following dimensions: Task, Authority, Recognition, Group, Evaluation, and Time	Educational attainment
Yildiz and Duy (2013)	Turkey	Controlled before versus after	16	Interpersonal communication skills training	Educational attainment

^a^ Outcomes related to domains other than education are not listed here.

##### Participant characteristics and intervention setting

###### Target group

**Figure 3:**
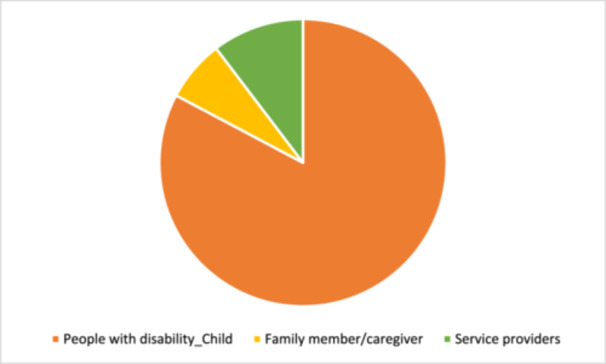
Target group.

Most studies (*n* = 25) targeted children with disabilities. Only two directly targeted family members, and three targeted service providers (see Figure 3 below).^3^ However, there were several interventions which were primarily delivered via family members and service providers. In these cases, while the child was still the intervention *target*, the family member of service provider was the intervention recipient, but this was not evaluated with a formal outcome measurement.

The socioeconomic status (SES) of participants was extremely poorly reported, with 21 studies failing to report on this. Most studies included both male and female participants (*n* = 19). Two studies included only male participants. In seven cases, it was not possible to tell the gender of the participants.

**Figure 4:**
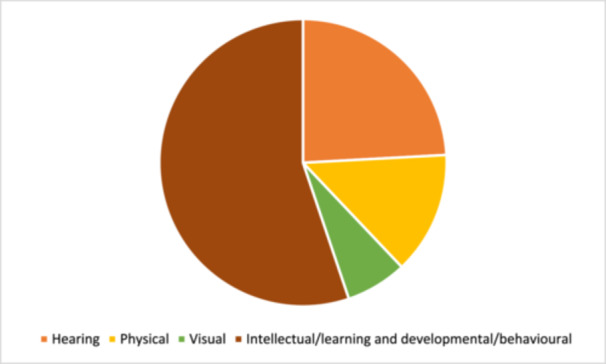
Participants by impairment type.

Individuals with intellectual or learning and developmental impairments were the most frequently targeted by included interventions (*n* = 16). This was followed by individuals with hearing impairments (*n* = 7), physical impairments (*n* = 4), and then vision impairments (*n* = 2). No studies identified in our review captured education interventions for people with psychosocial/mental impairments. Figure 4 below provides a visual summary of this distribution.

###### Location

**Figure 5:**
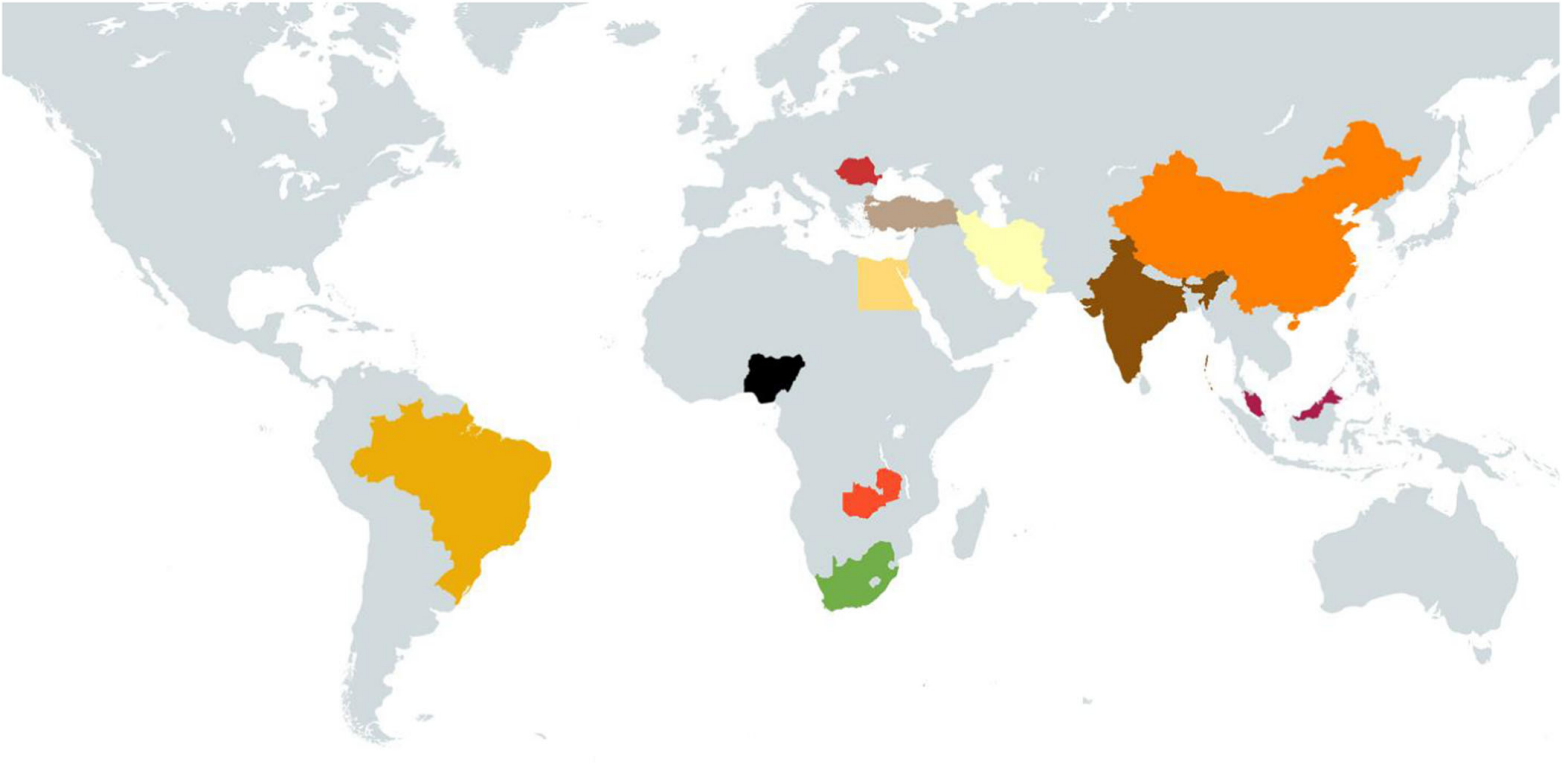
Countries of included studies.

Eight of the included studies were from India, four were from Iran, two were from China and two were from South Africa and Egypt, each. Brazil, Turkey, Jordan, Kenya, Lebanon, Romania, Malaysia, Nigeria, Uganda, and Zambia each contributed one study (see Figure 5 below). In terms of World Bank regions, two studies came from East Asia and the Pacific, one from Latin America and the Caribbean, nine from the Middle East and North Africa, six from Sub‐Saharan Africa, eight from South Asia, and one from Europe and Central Asia.

In many cases (*n* = 13), it was not possible to determine whether the study was conducted in a rural or urban setting. In 14 studies, urban participants were included, and in one study, the participants came from a mix of rural and urban settings. There were no studies which specifically targeted rural participants.

Table 5 below presents the intervention and outcome details of included studies (Table 6).

**Table 5:** Summary of findings by study.

Study	Design	Outcome domain	Confidence in study findings	Specific outcomes	Unit of effect	**Effect size** a
Adnams et al. (2007)	RCT	Educational attainment	High	All sounds	Standardised mean difference	0.54
				Blending syllables	Standardised mean difference	0.43
				Blending Phonemes	Standardised mean difference	1.32
				Manipulating syllables	Standardised mean difference	0.73
				Manipulating Phonemes	Standardised mean difference	0.45
				Letter sounds	Standardised mean difference	1.2
				Written letters	Standardised mean difference	1.02
				Reading	Standardised mean difference	1.21
				Spelling	Standardised mean difference	1.35
Akbari et al. (2019)	Controlled before versus after	Educational attainment	Low	Planning and organising	*T*‐test	*p* < 0.05^b^
				Inhibition and attention, displacement	*T*‐test	*p* < 0.05^b^
				Inhibition and attention	*T*‐test	*p* < 0.05
				Working memory	Standardised mean difference	0.93
				Reading	Standardised mean difference	3.83
Altakhyneh (2019)	Controlled before versus after	Educational attainment	Low	Mathematical performance	ANOVA	*p* < 0.001^b^
Awada and Gutiérrez‐Colón (2017)	Controlled before versus after	Educational attainment	Low	Comprehension of narrative texts (Grade 7)	Standardised mean difference	1.49
				Comprehension of narrative texts (Grade 8)	Standardised mean difference	1.78
				Comprehension of narrative texts (Grade 9)	Standardised mean difference	0.46
				Comprehension of narrative texts (Grade 10)	Standardised mean difference	0.81
Carew et al. (2019)	Uncontrolled before versus after	Accessible learning environments; Attendance and completion	Low	Teaching self‐efficacy	Standardised mean difference	2.20
				Beliefs	Standardised mean difference	0.69
				Feelings	Standardised mean difference	0.70
				Intentions	Standardised mean difference	0.22
				Self‐focused concerns	Standardised mean difference	**−**0.62
				Other‐focused concerns	Standardised mean difference	−0.95
Costescu et al. (2015)	Controlled before versus after	Educational attainment	Low	Errors in learning phase	Standardised mean difference	0.87
				Perseverative errors	Standardised mean difference	0.51
				Shared attention	Standardised mean difference	0.98
				Positive affect frequency	Standardised mean difference	0.44
				Regressive errors	Standardised mean difference	0.27
Devries et al. (2018)	RCT	Accessible learning environments	Low	Any violence, staff or peers, past week	aOR	*p* = 0.077^b^
				Any violence, staff or peers, past term	aOR	*p* = 0.223^b^
				Any staff violence, past week	aOR	*p* = 0.342^b^
				Any violence, staff, past term	aOR	*p* = 0.173^b^
				Physical violence from staff, past week	aOR	*p* = 0.427^b^
				Physical violence from staff, past term	aOR	*p* = 0.481^b^
				Any peer violence, past week	aOR	*p* = 0.073^b^
				Any peer violence, past term	aOR	*p* = 0.117^b^
Eissa (2009)	Controlled before versus after	Educational attainment	Low	Writing	Standardised mean difference	4.98
Hatamizadeh et al. (2020)	RCT	Educational attainment	Low	Resilience	*T*‐test	*p* < 0.001^b^
Johnson (2018)	Controlled before versus after	Educational attainment	Low	Visual motor integration	Standardised mean difference	1.29
				Visual perception	Standardised mean difference	1.13
				Motor Coordination	Standardised mean difference	2.15
				Word legibility	Standardised mean difference	0.11
				Letter legibility	Standardised mean difference	1.3
				Numeral legibility	Standardised mean difference	0.57
Karahmadi et al. (2014)		Educational attainment	Low	Speed reading	Standardised mean difference	0.20
				Reading accuracy	Standardised mean difference	0.07
				Percent of spelling scores	Standardised mean difference	0.09
Karande et al. (2007)	Uncontrolled before versus after	Accessible learning environments	Low	Parental knowledge of meaning of the term specific learning disabilities	Standardised mean difference	2.66
				Parental knowledge of remedial education given by specialist	Standardised mean difference	1.52
				Meaning of the term remedial education	Standardised mean difference	2.73
				Parental knowledge of frequency and duration of remedial education necessary to achieve academic competence	Standardised mean difference	2.86
				Parental knowledge of meaning and purpose of provisions	Standardised mean difference	2.3
				Parental knowledge about cause of specific learning disabilities	Standardised mean difference	0.73
Katongo and Ndhlovu (2015)	Controlled before versus after	Educational attainment	Low	Word intelligibility	Standardised mean difference	0.9
				Short sentence construction	Standardised mean difference	1.35
Kaur et al. (2008)	Controlled before versus after	Educational attainment	Low	Mathematical skills: Control v Group 1	Standardised mean difference	6.62
				Mathematical skills: Control v Group 2	Standardised mean difference	4.32
				Mathematical skills Control v Group 3	Standardised mean difference	6.93
Kumar and Chaturvedi (2014)	Controlled before versus after	Educational attainment	Low	Eye‐hand Coordination	Standardised mean difference	1.66
				Figure‐Ground Perception	Standardised mean difference	1.03
				Figure Constancy	Standardised mean difference	2.13
				Position in Space	Standardised mean difference	1.7
				Spatial Relation	Standardised mean difference	1.6
				Auditory Perception	Standardised mean difference	1.37
				Cognitive Abilities	Standardised mean difference	1.36
				Memory	Standardised mean difference	1.11
				Receptive Language	Standardised mean difference	1.29
				Expressive Language	Standardised mean difference	0.21
Lal and Bali (2007)	Controlled before versus after	Educational attainment	Low	Composite score	Mean difference	*p* > 0.05^b^
				Expressive language	Mean difference	*p* > 0.05^b^
				Receptive language	Mean difference	*p* > 0.05^b^
Lal (2010)	Uncontrolled before versus after	Educational attainment	Low	Social communicative behaviour	Wilcoxon Signed Rank Test	<0.01
				Responsive and expressive language	Wilcoxon Signed Rank Test	<0.01
Lal and Ganesan (2011)	Controlled before versus after	Educational attainment	Low	Rating scale for self‐management (RSSM): Classroom behaviour	*T*‐test	*p* < 0.001
Lee et al. (2019)	Uncontrolled before versus after	Educational attainment	Low	Adaptive behaviour (including socialisation, communication)	Standardised mean difference	−0.63
				Behavioural and functioning (including school functioning)	Standardised mean difference	0.96
Martin et al. (2001)	Uncontrolled before versus after	Educational attainment	Low	Problem‐solving skills, critical thinking	ANOVA	*p* < 0.003^b^
				Creative thinking	ANOVA	*p* > 0.05^b^
Mohammed and Kanpolat (2010)	Controlled before versus after	Educational attainment	Low	Classification skills	Standardised mean difference	9.86
Pawar and Mohite (2014)	Uncontrolled before versus after	Learning social environment and social inclusion	Low	Knowledge score	Standardised mean difference	7.65
Rezaiyan et al. (2007)	Controlled before versus after	Educational attainment	Low	Attention score	Standardised mean difference	0.78
Thai and Mohd Yasin (2016)	Controlled before versus after	Educational attainment	Low	Multiplication achievement	Standardised mean difference	0.49
Twilhaar (2012)	Uncontrolled before versus after	Educational attainment	Low	Reaching	Multiple regression coefficient	*p* < 0.01^b^
				Grasping	Multiple regression coefficient	*p* < 0.05^b^
				Gross motor level	Multiple regression coefficient	*p* > 0.05^b^
				Social responsiveness	Multiple regression coefficient	*p* > 0.05^b^
				Cognitive play performance	Multiple regression coefficient	*p* > 0.05^b^
Ugwuanyi and Adaka (2015)	Uncontrolled before versus after	Educational attainment	Low	Reading	Standardised mean difference	3.14
Valentini and Rudisill (2004)	Controlled before versus after	Educational attainment	Low	Locomotor performance	Standardised mean difference	0.20
Yildiz and Duy (2013)	Controlled before versus after	Educational attainment	Low	Empathy	*f*‐test	*p* = 0.323^b^
				Communication skills	*f*‐test	*p* = 0.824^b^

^a^ Standardised effect sizes are presented, except for cases where information necessary to calculate them was missing from the publication. In these latter instances, author‐reported *p*‐values are given.

^b^ Author‐reported *p*‐values.

**Table 6:** Summary of findings by outcome.

Outcome domain	Outcome sub‐category	Specific outcome	Number of participants
Educational attainment	Skills for formal/learning in schools	Reading ability (Adnams et al., 2007; Akbari et al., 2019; Awada & Gutiérrez‐Colón, 2017; Karahmadi et al., 2014; Ugwuanyi & Adaka, 2015) Numeracy and mathematical ability (Adnams et al., 2007; Altakhyneh, 2019; Elmonayer, 2017; Kaur et al., 2008) Spelling (Adnams et al., 2007; Karahmadi et al., 2014) Handwriting performance (Eissa, 2009; Johnson, 2018) Communication skills (Katongo & Ndhlovu, 2015; Lal & Bali, 2007; Lal, 2010) Remediation of learning disability [sic] (Kumar & Chaturvedi, 2014) Reasoning and classification skills (Martin et al., 2001; Mohammed & Kanpolat, 2010) Critical and creative thinking (Martin et al., 2001) Cognitive and executive functioning (Akbari et al., 2019; Costescu et al., 2015; Martin et al., 2001; Twilhaar, 2012) Attention (Rezaiyan et al., 2007) Classroom behaviour (Lal & Ganesan, 2011) Mathematic skill readiness (Kaur et al., 2008)	65 (Adnams et al., 2007) 20 (Akbari et al., 2019) 60 (Altakhyneh, 2019) 298 (Awada & Gutiérrez‐Colón, 2017) 81 (Costescu et al., 2015) 67 (Eissa, 2009) 5 (Elmonayer, 2017) 34 (Johnson, 2018) 52 (Karahmadi et al., 2014) 60 (Katongo & Ndhlovu, 2015) 40 (Kaur et al., 2008) 64 (Kumar & Chaturvedi, 2014) 30 (Lal & Bali, 2007) 8 (Lal, 2010) 20 (Lal & Ganesan, 2011) 47 (Martin et al., 2001) 68 (Mohammed & Kanpolat, 2010) 60 (Rezaiyan et al., 2007) 70 (Thai & Mohd Yasin, 2016) 36 (Twilhaar, 2012) 33 (Ugwuanyi & Adaka, 2015) 104 (Valentini & Rudisill, 2004)
	Skills for life	Social responsiveness (Twilhaar, 2012) Motor function (Twilhaar, 2012) Behaviour and social skills (Lal & Ganesan, 2011; Lal, 2010; Lee et al., 2019) Speech intelligibility (Katongo & Ndhlovu, 2015) Communication skills (Lal & Bali, 2007; Lal, 2010; Yildiz & Duy, 2013) Emotional skills (Lee et al., 2019; Yildiz & Duy, 2013) Attention (Costescu et al., 2015; Rezaiyan et al., 2007) Resilience (Hatamizadeh et al., 2020) Motor function (Twilhaar, 2012; Valentini & Rudisill, 2004)	81 (Costescu et al., 2015) 122 (Hatamizadeh et al., 2020) 60 (Katongo & Ndhlovu, 2015) 30 (Lal & Bali, 2007) 8 (Lal, 2010) 20 (Lal & Ganesan, 2011) 8 (Lee et al., 2019) 60 (Rezaiyan et al., 2007) 36 (Twilhaar, 2012) 104 (Valentini & Rudisill, 2004) 16 (Yildiz & Duy, 2013)
Accessible learning environments	Strengthened learning social environment and improved social inclusion	Teacher beliefs, feelings, and intentions about inclusive education [52] Teacher concerns about inclusive education [52] Parental knowledge, attitudes, and practices about remedial education (Karande et al., 2007)	130 (Carew et al., 2019) 50 (Karande et al., 2007) 60 (Pawar & Mohite, 2014)
	Reduced rates of bullying/victimisation in educational setting	Physical violence from a school staff member (Devries et al., 2018)	3820 (Devries et al., 2018)
	Educational services development	Teaching self‐efficacy (Carew et al., 2019) Knowledge of Primary School Teachers Regarding Learning Disorders (Pawar & Mohite, 2014)	130 (Carew et al., 2019) 60 (Pawar & Mohite, 2014)

##### Study characteristics

###### Study design

Of the 28 studies included, four were RCTs. 16 were controlled (before vs after), and the remaining eight were uncontrolled (before vs after).

###### Intervention characteristics

About a third of the included interventions were delivered by specialists, therapists, or intervention coaches (*n* = 11), and about a third by community members or existing staff (*n* = 10). In the rest of the interventions (*n* = 7), it was unclear who was responsible for delivery. Intervention settings were most commonly schools and classrooms in mainstream or inclusive settings, followed by specialist school or resource rooms in inclusive schools. The rest of the interventions were implemented in learning disorder centres, Organisations of Persons with Disabilities, or in care facilities.^4^ In three studies, the site of intervention was not reported (see Table 7).

**Table 7:** Intervention setting.

Schools and classrooms (inclusive)	13
Learning disorder centres	2
Specialist schools and resource rooms	8
Organisations of persons with disabilities	1
Care facility	1
Not reported	3

Regarding intervention content, coding was conducted according to the primary and, where there was one, secondary focus of the intervention.^5^ All coding categories were not mutually exclusive and so multiple coding was done where an intervention covered more than one category of intervention.

Across all studies, the category of interventions most commonly represented was ‘Educational attainment support’ (*n* = 28), followed by ‘Accessible learning environments’ (*n* = 16). Intervention categories were not mutually exclusive, given that some programmes were multi‐component. Nonetheless, when mapped against our intervention categories (see Table 1 above), none of the interventions were coded as focusing on ‘Attendance, enrolment, and completion’.

The types of interventions which fell under ‘Educational attainment support’ included, for instance, a reading comprehension intervention that employed combined strategy instruction (graphic organisers, visual displays, mnemonic illustrations, computer exercises, predicting, inference, text structure awareness, main idea identification, summarisation, and questioning) for children with dyslexia (Awada & Gutiérrez‐Colón, 2017). Indeed, most of these interventions focused on testing specific strategies – such as ‘drilling in singing’ [sic] (Katongo & Ndhlovu, 2015), multimedia, cognitive strategies and eclectic approaches (Kaur et al., 2008), and systematic cognitive‐strategy instruction (Martin et al., 2001) – in the context of either specialised or mainstream classes, with a view to improving learning outcomes among children with disabilities.

Within ‘Educational attainment support’, the interventions could be organised according to sub‐categories. The frequency of each sub‐category is presented in Table 8 below.

**Table 8:** Sub‐categories of ‘Educational attainment support’ intervention category.

Skills for formal/learning in schools	20
Skills for life	9
Education‐related quality of life	0

Clearly, many interventions focused on core competencies that could equip children to function in formal learning environments, but also in their broader environment(s). Examples of such programmes include the conductive education intervention documented in Twilhaar (2012), which aimed to teach and motivate children with cerebral palsy to participate and function in a range of activities including those relevant to classroom learning along with more general skills, such as independent eating. Another example of an outcome spanning both skills for formal learning and skills for life can be seen in the intervention evaluated by Rezaiyan et al. (2007), which targeted attentional capacity through a computer game.

However, in most studies, there was a focus on developing children's capacity to engage in *formal* learning within educational settings. These intervention components mostly centred on skills such as literacy (Adnams et al., 2007; Akbari et al., 2019; Awada & Gutiérrez‐Colón, 2017; Karahmadi et al., 2014; Ugwuanyi & Adaka, 2015) and numeracy (Adnams et al., 2007; Altakhyneh, 2019; Elmonayer, 2017; Kaur et al., 2008), or handwriting legibility (Eissa, 2009; Johnson, 2018). No interventions targeted education‐related quality of life.

Within the category of programming to improve ‘Accessible learning environments’, intervention components could be categorised according to the sub‐categories reflected in Table 9 below. The sub‐category most reflected among the interventions was ‘Learning social environment and social inclusion’. In other words, these interventions aimed to improve the quality and/or inclusiveness of learning social environments. Among programmes coded under this sub‐category were those that aimed to improve social skills (Lee et al., 2019) and reduce rates of victimisation of children with disabilities in school (Devries et al., 2018). Other sub‐categories reflected in the literature included ‘Structural interventions’ (*n* = 1) (Carew et al., 2019), ‘Anti‐bullying policies and programmes’ (*n* = 1) (Devries et al., 2018), ‘Educational services development’ (*n* = 6) (Carew et al., 2019; Pawar & Mohite, 2014), and ‘Rehabilitation and health services, and assistive technologies’ (Lal, 2010). No studies represented the intervention categories of ‘Accessibility of built environment and learning materials’ or ‘Inclusive education policies’.

**Table 9:** Sub‐categories of ‘Accessible learning environments’ intervention category.

Structural interventions	3
Learning social environment and social inclusion	7
Accessibility of built environment and learning materials	0
Anti‐bullying policies and programmes	1
Educational services development	6
Inclusive education policies	0
Rehabilitation and health services, and assistive technologies	1

##### Outcome characteristics

The category of outcome most frequently reflected in the studies was ‘Educational attainment’ (*n* = 31), followed by ‘Accessible learning environments’ (*n* = 6). Again, categories were not mutually exclusive, given that some programmes had multiple outcomes. The category ‘Attendance, enrolment, and completion’ was not represented among the outcomes.

As observed in these interventions, ‘Educational attainment’ outcomes could be broken down into those focused on ‘Skills for formal/learning in schools’ (*n* = 22) and ‘Skills for life’ (*n* = 12), as shown in Table 10. Exemplary among skills for formal learning/learning in schools were outcomes such as comprehension of narrative texts (Awada & Gutiérrez‐Colón, 2017) and mathematical skills (Kaur et al., 2008). Those exemplary among the ‘Skills for life’ category were outcomes such as word intelligibility (Katongo & Ndhlovu, 2015), as well as problem‐solving skills and critical thinking (Martin et al., 2001). ‘Education‐related quality of life’ was not represented among the study outcomes.

**Table 10:** Sub‐categories of ‘Educational attainment’ outcome category.

Skills for formal/learning in schools	22
Skills for life	12
Education‐related quality of life	0

The outcome category of ‘Accessible learning environments’ reflected the sub‐categories shown in Table 11. The most frequently represented sub‐categories were ‘Strengthened learning social environment and improved social inclusion’ (Carew et al., 2019; Karande et al., 2007; Pawar & Mohite, 2014) and ‘Educational services development’ (Carew et al., 2019; Valentini & Rudisill, 2004). These were followed by ‘Reduced rates of bullying/victimisation in educational setting’ (Devries et al., 2018). Outcomes for the category of ‘Strengthened learning social environment and improved social inclusion’ included, for instance, improved teacher intentions to include children with disabilities in mainstream classes (Carew et al., 2019), while examples of ‘Educational services development’ can be seen in Carew et al.'s (2019) intervention, which was focused on teaching self‐efficacy. Finally, ‘Reduced rates of bullying/victimisation in educational setting’ was reported on by Devries and colleagues (Devries et al., 2018), who looked at past‐week physical violence from a school staff member as self‐reported by students with disabilities.

**Table 11:** Sub‐categories of ‘Accessible learning environments’ outcome category.

Strengthened learning social environment and improved social inclusion	3
Improved accessibility of built environment and learning materials	0
Reduced rates of bullying/victimisation in educational setting	1
Educational services developed	2
Provision and utilisation of rehabilitation and health services, and assistive technologies	0

The outcome category of ‘Attendance, enrolment, and completion’ was not represented among the outcomes of any studies.

Table 6 (above) presents a summary of the findings of this review, by outcome of interest.

#### 5.1.3 Excluded studies

The number of excluded studies is recorded in the PRISMA diagram above (Figure 2). Examples of excluded studies with the associated reason for exclusion are presented in Annex S1C. The most common reason for exclusion was an insufficient sample size (<5 participants).

An important note regarding the exclusions of this review concerns ECD interventions. When the protocol for review was first developed, the authors envisaged that preschool and ECD interventions would be included. However, once the review was undertaken, this posed two challenges:

1. The types of interventions and outcomes evaluated in many ECD interventions are not clearly ‘education’ interventions or outcomes; and
2. The ECD literature in respect of child development and developmental disabilities is well‐summarised elsewhere.

To further explain, most of the ECD literature examines behavioural and parenting interventions delivered by teachers, or teachers plus trainers, to young children, with the aim of ‘managing’ children's behaviour. While these interventions are sometimes framed as education interventions, they are just as often framed as health interventions. Moreover, while it is plausible that interventions focused on strengthening eye contact or reducing ‘problem behaviours’ [sic] may lead to better learning over time, the idea that these interventions, their aims, or their outcomes are ‘education‐focused’ is debatable. Finally, a large number of reviews have already examined the ECD literature (Emmers et al., 2021; Jeong et al., 2021; Kohli‐Lynch et al., 2019; Oono et al., 2013) so it is questionable whether any value would be added by including these studies in another evidence synthesis. As such, the decision was taken to exclude papers where the target group was children under 6 years of age. Nonetheless, we provide a brief summary of the identified ECD literature in Table 12 below.

**Table 12:** Interventions excluded due to child age.

	Intervention	Child age	Country	Outcome(s)
Elmonayer (2017)	Visual scaffolding intervention to promote number sense	Mean age = 5.4 years	Egypt	Number sense
Juneja	A parent‐based behavioural intervention programme	Mean age = 3.3 years	India	Child behaviour, Developmental Quotient, Social Maturity, Receptive language, Expressive language
Karaaslan and Mahoney (2013)	A responsive teaching intervention	Mean age = 4.6 years	Turkey	Maternal behaviour, Child behaviour
Karanth et al. (2010)	An indigenous early childhood development intervention	Age range = 2.2–5.5 years	India	Child behaviour
Pajareya and Nopmaneejumruslers (2011)	A parent‐based behavioural intervention programme	Age range = 2–6 years	Thailand	Functional emotional development, Child behaviour
Pajareya and Nopmaneejumruslers (2012)	A parent‐based behavioural intervention programme	Age range = 2–6 years	Thailand	Functional emotional development, Child behaviour
Sarouphim and Kassem (2020)	A family‐based early childhood development intervention	Age range = 1–3 years	Lebanon	Child development
Shin et al. (2009)	A parent‐based behavioural intervention programme	Age range = 3–6 years	Vietnam	Child behaviour

### 5.2 Confidence in study findings in included studies

Besides one study which had a high overall confidence rating (Adnams et al.), all other included studies had an overall rating of low confidence in study findings (see Table 13). The overall confidence in these studies is generally low, primarily due to the low rigour in design and execution. Masking was often not implemented, and attrition rates were not consistently reported, which affects the reliability of the findings. Disability and outcome measures varied in quality, with many studies failing to achieve a high rating. Baseline balance was also frequently low, indicating issues with the initial equivalence of groups. Collectively, these methodological shortcomings highlight the need for more rigorous research designs in future studies to provide more reliable and generalisable results.

**Table 13:** Confidence in study findings appraisal.

		Design			Masking			Attrition			Disability measure			Outcome measure			Baseline balance			Overall		
Study	Design	Low	Medium	High	Low	Medium	High	Low	Medium	High	Low	Medium	High	Low	Medium	High	Low	Medium	High	Low	Medium	High
Adnams et al. (2007)	RCT			●			●			●			●			●			●			●
Akbari et al. (2019)	Controlled before versus after	●								●			●			●	●			●		
Altakhyneh (2019)	Controlled before versus after	●								●		●				●	●			●		
Awada and Gutiérrez‐Colón (2017)	Controlled before versus after	●						●					●		●		●			●		
Carew et al. (2019)	Uncontrolled before versus after	●								●						●				●		
Costescu et al. (2015)	Controlled before versus after	●						●				●			●				●	●		
Devries et al. (2018)	RCT			●		●		●					●			●			●	●		
Eissa (2009)	Controlled before versus after	●								●			●			●		●		●		
Hatamizadeh et al. (2020)	RCT			●			●	●				●				●			●	●		
Johnson (2018)	Controlled before versus after	●								●			●			●	●			●		
Karahmadi et al. (2014)	RCT			●	●					●			●			●			●	●		
Karande et al. (2007)	Uncontrolled before versus after	●								●			●			●				●		
Katongo and Ndhlovu (2015)	Controlled before versus after	●								●		●				●	●			●		
Kaur et al. (2008)	Controlled before versus after	●								●			●			●	●			●		
Kumar and Chaturvedi (2014)	Controlled before versus after	●								●			●			●	●			●		
Lal and Bali (2007)	Controlled before versus after	●								●			●			●	●			●		
Lal (2010)	Uncontrolled before versus after	●								●			●			●				●		
Lal and Ganesan (2011)	Controlled before versus after	●						●				●				●	●			●		
Lee et al. (2019)	Uncontrolled before versus after	●								●		●	●			●				●		
Martin et al. (2001)	Uncontrolled before versus after	●						●			●					●				●		
Mohammed and Kanpolat (2010)	Controlled before versus after	●								●			●			●		●		●		
Pawar and Mohite (2014)	Uncontrolled before versus after	●						●						●						●		
Rezaiyan et al. (2007)	Controlled before versus after	●						●			●			●				●		●		
Thai and Mohd Yasin (2016)	Controlled before versus after	●								●			●			●		●		●		
Twilhaar (2012)	Uncontrolled before versus after	●						●			●					●				●		
Ugwuanyi and Adaka (2015)	Uncontrolled before versus after	●								●		●				●				●		
Valentini and Rudisill (2004)	Controlled before versus after	●						●			●		●		●		●			●		
Yildiz and Duy (2013)	Controlled before versus after	●						●				●				●	●			●		

*Note*: Grey blocks indicate where a criterion was not applicable to a study.

As our assessment tool assigned studies an overall rating based on the lowest rating on any criteria, we discuss confidence in study findings by domain below, to present a more nuanced picture of quality and risk of bias in the included literature.

Most low rated studies received a rating that led to their overall downgrading due to their study design. Twenty‐four studies (Akbari et al., 2019; Altakhyneh, 2019; Awada & Gutiérrez‐Colón, 2017; Carew et al., 2019; Costescu et al., 2015; Eissa, 2009; Johnson, 2018; Karande et al., 2007; Katongo & Ndhlovu, 2015; Kaur et al., 2008; Kumar & Chaturvedi, 2014; Lal & Bali, 2007; Lal & Ganesan, 2011; Lal, 2010; Lee et al., 2019; Martin et al., 2001; Mohammed & Kanpolat, 2010; Pawar & Mohite, 2014; Rezaiyan et al., 2007; Thai & Mohd Yasin, 2016; Twilhaar, 2012; Ugwuanyi & Adaka, 2015; Valentini & Rudisill, 2004; Yildiz & Duy, 2013) were categorised as low on design, while only four were categorised as high (Adnams et al., 2007; Devries et al., 2018; Hatamizadeh et al., 2020; Karahmadi et al., 2014). Low ratings were chiefly given because of the use of controlled or uncontrolled before‐versus‐after designs. The four high confidence ratings were given for RCTs.

In the cases where reporting of masking was applicable (the RCTs), one of these studies was rated as medium (Devries et al., 2018), for motivating that the failure to mask was due to the nature of the intervention. Two were rated as high (Adnams et al., 2007; Hatamizadeh et al., 2020), for reporting masking of data collection and masking for analysis. One study (Karahmadi et al., 2014) received a low rating for failure to report on masking.

Ratings for attrition were based on losses to follow up being presented and acceptable. 11 studies received low ratings on loss to follow up (Awada & Gutiérrez‐Colón, 2017; Costescu et al., 2015; Devries et al., 2018; Hatamizadeh et al., 2020; Lal & Ganesan, 2011; Martin et al., 2001; Pawar & Mohite, 2014; Rezaiyan et al., 2007; Twilhaar, 2012; Valentini & Rudisill, 2004; Yildiz & Duy, 2013), while 17 were rated as high (Adnams et al., 2007; Akbari et al., 2019; Altakhyneh, 2019; Carew et al., 2019; Johnson, 2018; Karahmadi et al., 2014; Karande et al., 2007; Katongo & Ndhlovu, 2015; Kaur et al., 2008; Kumar & Chaturvedi, 2014; Lal & Bali, 2007; Lal, 2010; Lee et al., 2019; Mohammed & Kanpolat, 2010; Thai & Mohd Yasin, 2016; Ugwuanyi & Adaka, 2015). Low ratings were generally given due to a failure to report attrition.

For the clarity and reliability of the disability/impairment measure used in reference to the target group in studies, four papers received a rating of low (Martin et al., 2001; Rezaiyan et al., 2007; Twilhaar, 2012; Valentini & Rudisill, 2004), eight received a medium rating (Altakhyneh, 2019; Costescu et al., 2015; Hatamizadeh et al., 2020; Katongo & Ndhlovu, 2015; Lal & Ganesan, 2011; Lee et al., 2019; Ugwuanyi & Adaka, 2015; Yildiz & Duy, 2013), and the rest were rated as high (Adnams et al., 2007; Akbari et al., 2019; Awada & Gutiérrez‐Colón, 2017; Devries et al., 2018; Eissa, 2009; Johnson, 2018; Karahmadi et al., 2014; Karande et al., 2007; Lee et al., 2019; Mohammed & Kanpolat, 2010; Thai & Mohd Yasin, 2016; Valentini & Rudisill, 2004). For two studies, no rating was given as the target group of intervention was teachers without disabilities (Carew et al., 2019; Pawar & Mohite, 2014).

To aid interpretation and reliability of findings for comparability with other studies, outcome measures must be clearly defined. Against this criterion, two studies received a low rating (Pawar & Mohite, 2014; Rezaiyan et al., 2007), three received ratings of medium (Awada & Gutiérrez‐Colón, 2017; Costescu et al., 2015; Valentini & Rudisill, 2004), and the rest received ratings of high (Adnams et al., 2007; Akbari et al., 2019; Altakhyneh, 2019; Carew et al., 2019; Devries et al., 2018; Eissa, 2009; Hatamizadeh et al., 2020; Johnson, 2018; Karahmadi et al., 2014; Karande et al., 2007; Katongo & Ndhlovu, 2015; Kaur et al., 2008; Kumar & Chaturvedi, 2014; Lal & Bali, 2007; Lal & Ganesan, 2011; Lal, 2010; Lee et al., 2019; Martin et al., 2001; Mohammed & Kanpolat, 2010; Thai & Mohd Yasin, 2016; Twilhaar, 2012; Ugwuanyi & Adaka, 2015; Yildiz & Duy, 2013). In the case of these high ratings, studies had reported the outcomes being used (with a definition) and had provided the basis on which they were measured, often employing widely used and validated measures.

Of the 20 studies for which baseline balance was applicable for assessment (excluding uncontrolled before vs. after studies), 11 received low ratings (Akbari et al., 2019; Altakhyneh, 2019; Awada & Gutiérrez‐Colón, 2017; Johnson, 2018; Katongo & Ndhlovu, 2015; Kaur et al., 2008; Kumar & Chaturvedi, 2014; Lal & Bali, 2007; Lal & Ganesan, 2011; Valentini & Rudisill, 2004; Yildiz & Duy, 2013), 4 received medium ratings (Eissa, 2009; Mohammed & Kanpolat, 2010; Rezaiyan et al., 2007; Thai & Mohd Yasin, 2016), and 5 received a high rating (Adnams et al., 2007; Costescu et al., 2015; Devries et al., 2018; Hatamizadeh et al., 2020; Karahmadi et al., 2014).

### 5.3 Effects of interventions

**Figure 6:**
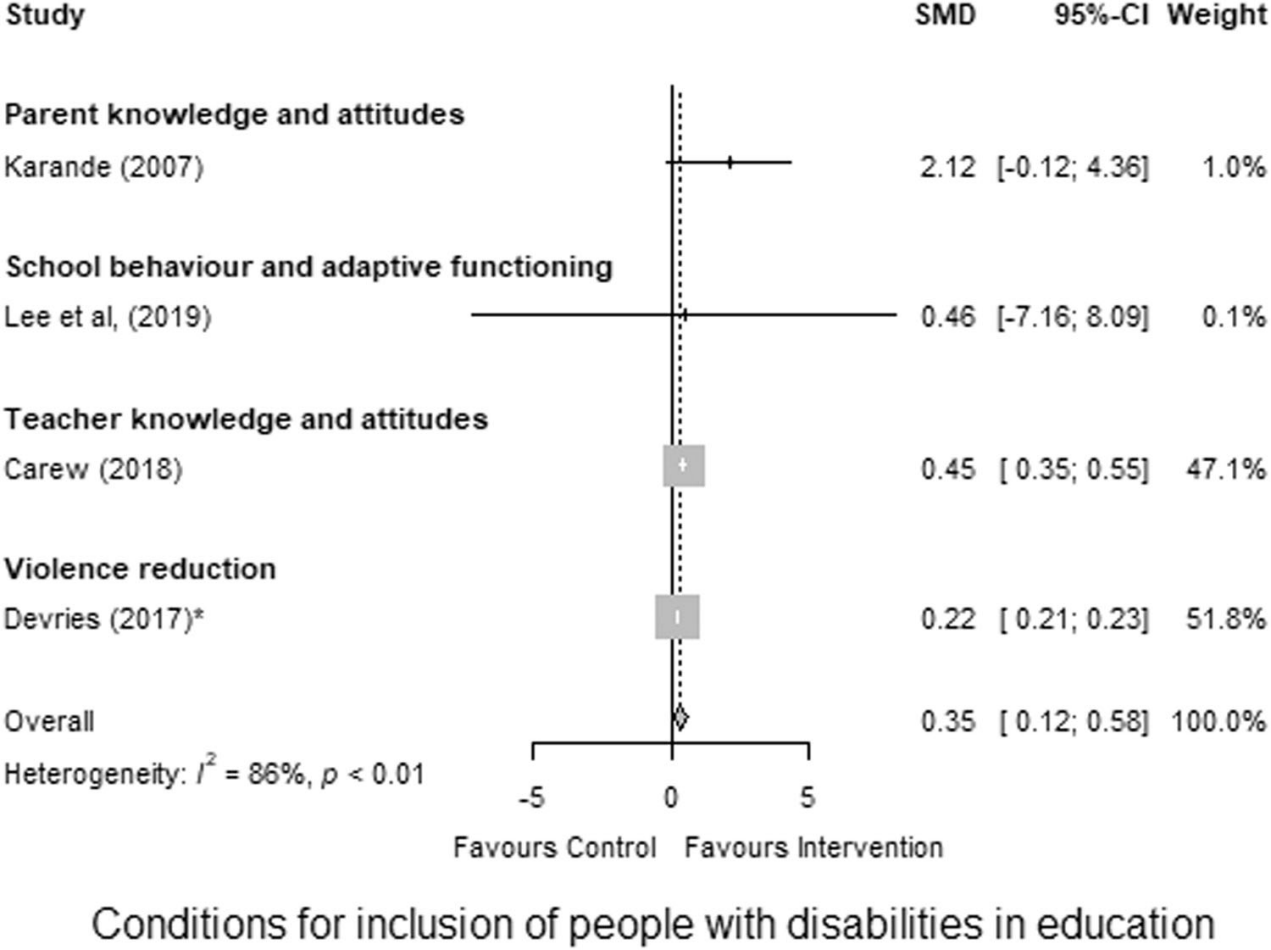
Overall effect size of interventions concerned with conditions for inclusion.

**Figure 7:**
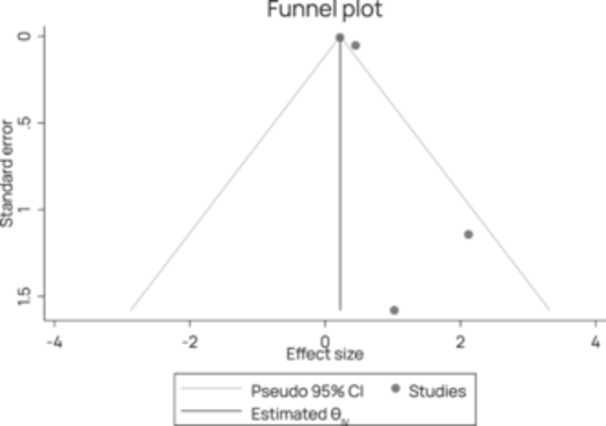
Potential bias in studies concerned with conditions for inclusion.

Included studies concerned with ‘Conditions for inclusion of people with disabilities in education’ showed a moderately significant effect, and one study concerned with teacher knowledge showed a significant effect size (see Figure 6). However, these studies showed a large heterogeneity and a potential publication bias (see Figure 7). The main limitation in this case was the low number of studies concerned with this topic (Table 14).

**Table 14:** Effects of interventions.

	Outcome	Effect	Summary
Conditions for inclusion of people with disabilities in education		*d* = 0.35 (0.12 to 0.58) *k* = 4 *n* = 401 I² = 86% Egger's test 2.01 (*t* = 1.43, *p* = 0.29)	Moderate effect based on a low number of studies with high heterogeneity and potentially moderate publication bias
Skills for learning	Speech and communication	*d* = 0.70 (−1.70 to 3.09) *k* = 5 *n* = 245 *I*² = 90% Egger's test 0.25 (*t* = 0.09, *p* = 0.93)	Insignificant effect based on a low number of studies with high heterogeneity and no publication bias
	School behaviour and adaptive functioning	*d* = 0.49 (−1.76 to 2.74) *k* = 5 *n* = 256 *I*² = 0% Egger's test 0.11 (*t* = 1.03, *p* = 0.38)	Insignificant effect based on a low number of studies with low heterogeneity and no publication bias
	Numeracy	*d* = 4.60 (0.69 to 8.50) *k* = 2 *n* = 110 *I*² = 85% Egger's test *Low sample size*	High effect based on a low number of studies with high heterogeneity. The publication bias cannot be assessed due to low sample size
	Literacy	*d* = 2.46 (0.01 to 4.90) *k* = 4 *n* = 389 *I*² = 93% Egger's test 3.61 (*t* = 1.66, *p* = 0.24)	Large effect based on a low number of studies with high heterogeneity and potentially high publication bias
	Handwriting	*d* = 4.86 (0.25 to 9.48) *k* = 2 *n* = 101 *I*² = 0% Egger's test *Low sample size*	Large effect based on a low number of studies with low heterogeneity. The publication bias cannot be assessed due to low sample size
	Cognitive skills, including attention and memory	*d* = 3.38 (0.96 to 5.80) *k* = 4 *n* = 212 *I*² = 100% Egger's test 15.30 (*t* = 0.99, *p* = 0.43)	Large effect based on a low number of studies with high heterogeneity and potentially high publication bias
	Overall	*d* = 2.10 (0.91 to 3.29) *k* = 22 *n* = 1313 *I*² = 99% Egger's test 5.24 (*t* = 1.93, *p* = 0.07)	Large effect based on a large number of studies with high heterogeneity and marginally significant publication bias

*Note*: *d* < 0.2 small, 0.2 < *d* < 0.6 moderate and *d* > 0.6 large. *k* < 10 small, 10 ≤ *k* < 20 moderate and *k* ≥ 20 large (*k* = number of studies). *I*² < 0.4 low, 0.4 ≤ *I*² < 0.8 moderate and *I*² ≥ 0.8 high. *n* = total number of participants.

**Figure 8:**
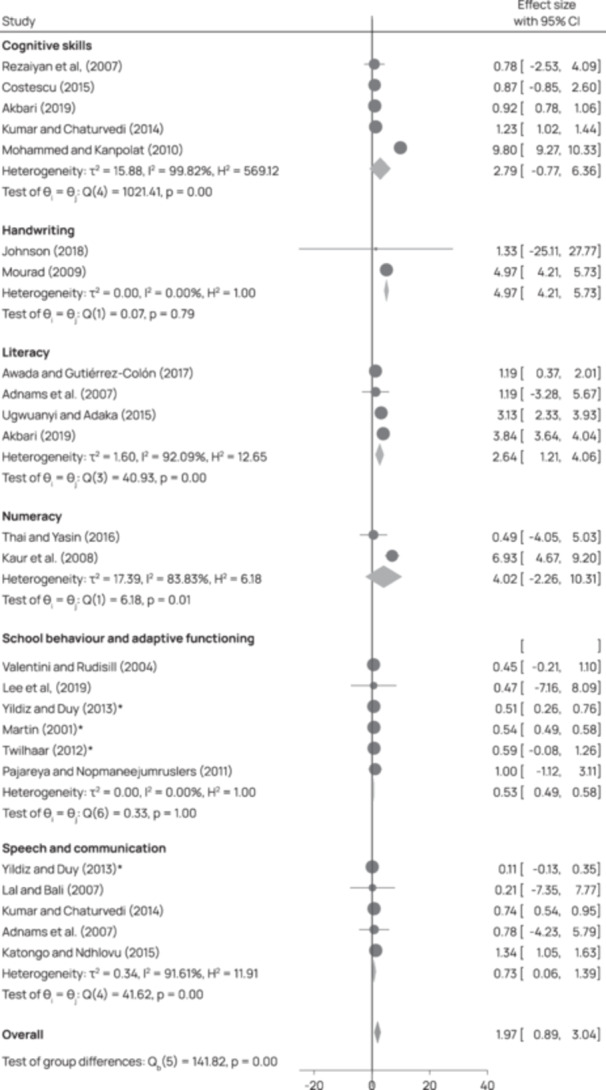
Overall effect of the intervention(s) on skills for learning.

The forest plot (Figure 8) below displays the overall effect of the intervention on ‘Skills for learning’. The forest plot also indicates that interventions tended to have a large effect on skills, but that there was high heterogeneity across published sources and sample sizes tended to be low. The joint effect was significant, at an alpha level of 5%, but it presents considerable heterogeneity and a marginally significant publication bias.

When considering the effect of intervention on different outcomes, we see that the effect on cognitive skills, numeracy, speech and communication is insignificant. The studies concerned with speech and school behaviour show no significant effect of intervention. In both cases, only 2 out of 5 studies showed an effect size different from 0. Literacy interventions demonstrate moderate effect but high heterogeneity (*I*² = 92.09%), with an effect size of 2.64 (CI: [1.21, 4.06]). Handwriting exhibits a significant and a large effect of 4.97 (CI: [4.21, 5.73]) with low heterogeneity (*I*² = 0.00%) but this is based on only two studies. The overview of the funnel plot (Figure 9) likewise shows that the former two categories are likely to suffer from publication bias, even though the Eggers Test is insignificant, due to a low sample size. The publication bias for numeracy and handwriting cannot be assessed, as the number of studies is only two.

### 5.4 Synthesis of results

**Figure 9:**
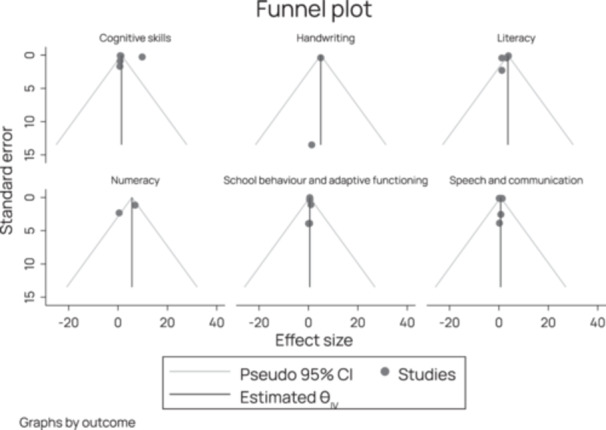
Potential bias in interventions concerned with skills for learning.

## 6 DISCUSSION

### 6.1 Summary of main results

We identified, coded, analysed, and narratively summarised the findings from 28 studies that evaluated interventions to improve the educational outcomes of people with disabilities in LMICs. These 28 studies served as the data for this review and are reported on according to the interventions and outcomes identified across all studies.

In terms of target group, the vast majority of studies concerned interventions for individuals with intellectual, learning, and/or developmental conditions. Most interventions were undertaken with disabled children, with a few targeting service providers, teachers, or family. Almost all studies targeted both male and female participants.

If examined by World Bank region, there were eight studies from South Asia, eight from the Middle East and North Africa, six from Sub‐Saharan Africa, three from East Asia and the Pacific, two from Europe and Central Asia, and one from Latin America and the Caribbean. Geographic setting (urban/rural) was not reported in over half of the studies, and participant SES was poorly reported, with the vast majority of studies not reporting on it.

In terms of study characteristics, most of the included studies employed controlled before versus after designs, followed by uncontrolled before versus after designs. A minority of studies used an RCT design.

Overall, only one study received a rating of ‘high’ confidence and all other included studies received ratings of ‘low’ on the confidence in study findings tool. Low ratings were driven by the widespread use of low‐rigour study designs, as well as failures to report important information such as balance, masking, and attrition.

The interventions under study were mostly delivered in mainstream settings, but this majority was not huge. Thirteen interventions were conducted in (inclusive) mainstream settings, while 12 happened in segregated or specialised settings, and a further 3 did not report on their setting. Most interventions were delivered by specialists, therapists or intervention coaches, and the minority by community members or existing staff.

The outcomes most frequently reflected in the reviewed studies were those which concerned educational attainment. This was followed in frequency by studies which looked at improving the accessibility of learning environments. No study looked at outcomes related to attendance, enrolment, and completion of people with disabilities in education. No studies reported on outcomes such as qualifications gained, transition to higher levels of education, or education‐related quality of life.

Among those studies which aimed to improve educational attainment, positive effects of interventions were seen for speech and communication; literacy; numeracy; handwriting; cognitive skills, including attention and memory; and school behaviour and adaptive functioning. Studies that were aimed at improving the accessibility of learning environments in education reported positive impacts of these interventions on teacher knowledge and attitudes, parent knowledge and attitudes, and violence reduction. Finally, attempts to improve attendance, enrolment, and completion among people with disabilities by equipping mainstream settings for the attendance of children with disabilities, was achieved in one study, where teacher knowledge and attitudes about inclusive education were improved.

The interventions and outcome measures used by the included studies were all different, making direct comparison (e.g., across countries) difficult. Most interventions tried to improve children's skills but did not focus on system‐level or school‐level changes. None of the studies were undertaken in humanitarian settings, and while almost all were undertaken with both male and female participants, few disaggregated their findings by gender.

### 6.2 Overall completeness and applicability of evidence

The evidence presented here provides emerging support for the efficacy and effectiveness of interventions to improve the education outcomes among people with disabilities in LMICs. However, due to the broad variety of interventions and outcomes assessed under the domain of education, and the limited number of high‐quality RCT and quasi‐experimental studies available, more research is needed to understand which types of interventions are most efficacious, and how best to deliver them.

### 6.3 Quality of the evidence

The quality of the included studies is largely low, as determined by the confidence in study findings tool used in this review. Most study designs employed were unable to consider many potential confounders. Moreover, masking, baseline balance, and attribution were poorly reported.

### 6.4 Potential biases in the review process

Potential bias may be introduced about the lack of grey literature included in the review, as well as the absence of non‐English literature.

### 6.5 Agreements and disagreements with other studies or reviews

Our findings largely concur with other reviews which have been conducted in the area, especially in the need for additional and more rigorous studies to be conducted, and reporting quality to be improved.

From the Cochrane databases, a review by Pennington et al. (2018) assessed the effectiveness of parent‐mediated communication interventions, for improving the communication skills of preschool children up to 5 years of age who have non‐progressive motor disorders. Our review differed somewhat from the 2018 review in that we excluded ECD interventions (where the outcomes only pertained to child developmental progress on standardised measures), because we operationalised educational outcomes as different to those relating to global development and functioning. However, Pennington et al. also found that their conclusions were limited by the low quality, and general dearth, of evidence in this area. They called for research with larger numbers of children, and for improved reporting standards, both of which are echoed by the findings of our review.

A review of the evidence concerning music education for improving reading skills in children and adolescents with dyslexia by Cogo‐Moreira et al. (2012) found that there was no evidence available from RCTs to inform judgments about the effectiveness of these programmes. While we did not find any RCTs evaluating music education, we did identify a study by Katongo and Ndhlovu (2015) which examined the impact of a music intervention on speech intelligibility of learners with hearing impairments. However, the study was not an RCT and given that it stood alone and pooled analysis was not possible, we cannot draw any specific conclusions about this modality of intervention.

Another Cochrane review, which examined task‐oriented interventions for children with developmental co‐ordination disorder (Miyahara et al., 2017), found that while beneficial effects of interventions were reported across most studies, there was very little confidence to be had in a positive effect estimate. Like Pennington et al. (2018), Miyahara et al. (2017) also called for carefully designed and executed RCTs in this area.

Other more topic‐specific rigorous reviews have been conducted and reported in the peer‐reviewed literature (Buysse & Bailey, 1993; Elbaum et al., 1999; Forlin et al., 2013; Gersten et al., 2001; Hudson et al., 2013; Katz & Mirenda, 2002; Paradise et al., 2009; Pierce et al., 2004; Purdie et al., 2002; Reichrath et al., 2010; Ruijs & Peetsma, 2009; Trout et al., 2003; Wapling, 2016).

Reichrath et al. (2010) investigate the interventions used in general education and what is known about their effectiveness. They found that all of the eight reading interventions they found and reviewed seem to have had positive influences on reading skills. However, the methodological quality in some studies was low.

Ruijs and Peetsma (2009) examined the impacts of inclusive education environments on children with special education needs and children without special education needs. Their review showed neutral to positive effects of inclusive education on academic achievement. However, they noted that children with special educational needs seem to have a less favourable social outcomes in mainstream settings compared to children without special educational needs. Our review did not reveal any systematic differences between the effectiveness of interventions delivered in mainstream or segregated/specialised settings, but this was likely due to the majority of interventions reporting positive findings.

Wapling (2016) conducted a systematic review to examine strategies being used in education for children with disabilities in LMICs. Their review was not focused on impact evaluations only, so many of the findings are not comparable to those in our review. However, the authors also noted the absence of literature analysing outcomes related to academic achievement as a significant gap in the research. While our review identified some studies that looked at academic functioning, skills and competencies, we also failed to find any which reported on more finite outcomes, such as qualifications gained and successful transitions to higher levels of education.

Finally, Saran et al. (2020) conducted an EGM on interventions for people with disabilities in LMICs. Their EGM found that with regard to education, few studies reported on the participation of children with disabilities in formal education. The most commonly reported education outcome in their EGM was ‘social and life skills development’ with effects reported from health interventions (rehabilitation and promotion), as well as early child development, and non‐formal education. Our findings differed in this respect, but this difference is largely due to the inclusion and exclusion criteria employed in our systematic review as compared to the EGM. We excluded certain studies where the intervention and outcomes lacked a clear education focus, and so the rehabilitation interventions and social skills development outcomes included under education in White et al.'s EGM were excluded here and are instead dealt with in two other systematic reviews on health outcomes and social inclusion outcomes respectively, also by our team. The EGM also did not find many studies conducted with primary and secondary school‐aged participants, which we have included in this review. Again, this is due to different inclusion criteria, partly because the EGM included vocational training with livelihoods outcomes while this review did not.

## 7 AUTHORS' CONCLUSIONS

Children with disabilities fall behind in educational outcomes as the current school systems are not set up to teach children with different impairment types. There is no one ‘magic bullet’ intervention which can equalise health outcomes for this group. A twin‐track approach is needed, which both addresses the specific needs of children with disabilities but also ensures that they are included in mainstream activities (e.g., through improving the skills of teachers and accessibility of the classroom). However, currently most interventions included in this systematic review targeted individual children with disabilities in an attempt to improve their functioning, skills, and competencies, but did not focus on mainstreaming these children into the school by system‐level or school‐level changes. Consequently, a focus on interventions which target not just the individual with a disability but also their broader environment, is needed.

### 7.1 Implications for practice

- Most interventions tried to improve children's functioning, skills, and competencies, but did not focus on system‐level or school‐level changes, that is, changes at a wider institutional level. Interventions which target both the individual with a disability, such as provision of speech‐to‐text software and their broader environment(s), such as teacher training programmes and curriculum modifications, are needed.
- Efforts should also be made to integrate measures of disability within planned or ongoing mainstream education impact evaluations and other demographic or household surveys that include education outcomes.
- Relevant existing programmes (not disability targeted) which are being implemented by governments, disabled persons organisations and non‐governmental organisations, should evaluate whether they are effective in improving educational outcomes for people with disabilities.
- There should be a focus on interventions that not only improve individual skills but also address broader issues such as attendance, enrolment, completion, and transition to higher levels of education.

### 7.2 Implications for research

- There is a need for more research in underrepresented regions and settings, such as Latin America and the Caribbean, rural areas and humanitarian contexts, to address the current gaps in the literature.
- Studies should focus on a broader range of outcomes, including qualifications gained, transition to higher levels of education, and education‐related quality of life, to provide a fuller understanding of the impact of interventions.
- Research should explore the effectiveness of interventions that target system‐level changes, such as policy reforms and school infrastructure improvements, to support inclusive education.
- Researchers should ensure comprehensive reporting of study details, including participant characteristics, intervention components, and implementation fidelity, to facilitate replication and meta‐analyses.
- Interventions, where appropriate, also need to be evaluated in terms of outcomes such as qualifications gained and transition to higher levels of education.
- Impact evaluations need to be funded and undertaken on ‘what works to improve educational outcomes for people with disabilities’. Studies conducted with larger samples of children are needed, as are those employing more rigorous study designs. The use of standardised measures, where possible, would also benefit comparison between studies and across regions.
- Studies should consistently consider and report on a broad range of characteristics and aspects of identity (e.g., gender, ethnicity) to understand the differential impacts of interventions on male and female participants with disabilities or impact on different ethnicities.

## CONTRIBUTIONS OF AUTHORS

- Content: All
- Systematic review methods: XH, AS, HW
- Statistical analysis: AS
- Information retrieval: XH, AS

## DECLARATIONS OF INTEREST

The authors have no interests to declare.

## DIFFERENCES BETWEEN PROTOCOL AND REVIEW

There are few differences between the protocol and the review, aside from the exclusion of ECD studies (discussed above), and the refinement of the intervention and outcome categories.

## SOURCES OF SUPPORT

### 1 Internal sources

No sources of support provided.

### 2 External sources

This systematic review is supported by the UK Department of International Development (DFID) under its support for the Centre for Excellence for Development Impact and Learning (CEDIL) and the Programme for Evidence to iNform Disability Action (PENDA).

## Supporting information

Supporting information.

## Footnotes

1 Thanks to Hugh Waddington (3ie and Campbell IDCG) for their suggestions, which were used in developing the tool.

2 See Table 1 of the standards of the United States Institute of Education Sciences What Works Clearing House.

3 These categories are not mutually exclusive, as some interventions targeted both children and caregivers, or children and service providers.

4 In some instances, studies did not clearly state where the intervention itself was implemented. In these instances, the site from which the participants were recruited was taken to be the setting of the intervention.

5 An example of where this can be seen is in terms of the intervention domain ‘Rehabilitation and health services, and assistive technologies’. Only two studies were coded as being focused primarily on these domains. However, a total of 20 studies *included* assistive devices or rehabilitation components, but not to the degree that warranted the intervention being coded as an intervention for ‘Rehabilitation and health services, and assistive technologies’.
